# Small Molecule Inhibition of an Exopolysaccharide Modification Enzyme is a Viable Strategy To Block Pseudomonas aeruginosa Pel Biofilm Formation

**DOI:** 10.1128/spectrum.00296-23

**Published:** 2023-04-26

**Authors:** Erum Razvi, Benjamin R. DiFrancesco, Gregory A. Wasney, Zachary A. Morrison, John Tam, Anick Auger, Perrin Baker, Noor Alnabelseya, Jacquelyn D. Rich, Piyanka Sivarajah, Gregory B. Whitfield, Joe J. Harrison, Roman A. Melnyk, Mark Nitz, P. Lynne Howell

**Affiliations:** a Program in Molecular Medicine, The Hospital for Sick Children, Toronto, Ontario, Canada; b Department of Biochemistry, University of Toronto, Toronto, Ontario, Canada; c Department of Chemistry, University of Toronto, Toronto, Ontario, Canada; d The Structural & Biophysical Core Facility, The Hospital for Sick Children, Toronto, Ontario, Canada; e SPARC BioCentre, The Hospital for Sick Children, Toronto, Ontario, Canada; f Department of Biological Sciences, University of Calgary, Calgary, Alberta, Canada; Weizmann Institute of Science

**Keywords:** high-throughput screen, Pel polysaccharide, *Pseudomonas aeruginosa*, biofilms, exopolysaccharide, inhibitors

## Abstract

Biosynthesis of the Pel exopolysaccharide in Pseudomonas aeruginosa requires all seven genes of the *pelABCDEFG* operon. The periplasmic modification enzyme PelA contains a C-terminal deacetylase domain that is necessary for Pel-dependent biofilm formation. Herein, we show that extracellular Pel is not produced by a P. aeruginosa PelA deacetylase mutant. This positions PelA deacetylase activity as an attractive target to prevent Pel-dependent biofilm formation. Using a high-throughput screen (*n* = 69,360), we identified 56 compounds that potentially inhibit PelA esterase activity, the first enzymatic step in the deacetylase reaction. A secondary biofilm inhibition assay identified methyl 2-(2-pyridinylmethylene) hydrazinecarbodithioate (SK-017154-O) as a specific Pel-dependent biofilm inhibitor. Structure-activity relationship studies identified the thiocarbazate as a necessary functional group and that the pyridyl ring could be replaced with a phenyl substituent (compound 1). Both SK-017154-O and compound 1 inhibit Pel-dependent biofilm formation in Bacillus cereus ATCC 10987, which has a predicted extracellular PelA deacetylase in its *pel* operon. Michaelis-Menten kinetics determined SK-017154-O to be a noncompetitive inhibitor of PelA, while compound 1 did not directly inhibit PelA esterase activity. Cytotoxicity assays using human lung fibroblast cells showed that compound 1 is less cytotoxic than SK-017154-O. This work provides proof of concept that biofilm exopolysaccharide modification enzymes are important for biofilm formation and can serve as useful antibiofilm targets.

**IMPORTANCE** Present in more than 500 diverse Gram-negative and 900 Gram-positive organisms, the Pel polysaccharide is one of the most phylogenetically widespread biofilm matrix determinants found to date. Partial de-*N*-acetylation of this α-1,4 linked *N*-acetylgalactosamine polymer by the carbohydrate modification enzyme PelA is required for Pel-dependent biofilm formation in Pseudomonas aeruginosa and Bacillus cereus. Given this and our observation that extracellular Pel is not produced by a P. aeruginosa PelA deactylase mutant, we developed an enzyme-based high-throughput screen and identified methyl 2-(2-pyridinylmethylene) hydrazinecarbodithioate (SK-017154-O) and its phenyl derivative as specific Pel-dependent biofilm inhibitors. Michaelis-Menten kinetics revealed SK-017154-O is a noncompetitive inhibitor and that its noncytotoxic, phenyl derivative does not directly inhibit P. aeruginosa PelA esterase activity. We provide proof of concept that exopolysaccharide modification enzymes can be targeted with small molecule inhibitors to block Pel-dependent biofilm development in both Gram-negative and Gram-positive bacteria.

## INTRODUCTION

Bacterial biofilms are matrix-enclosed microbial communities composed of self-produced extracellular polymeric substances (EPSs) that adhere to each other and/or to biological and nonbiological surfaces. Many human bacterial infections have biofilm etiology. These infections are often highly recalcitrant to antimicrobial treatment. Surgery is often required to resolve infection (1, 2), resulting in prolonged hospital stays, delaying patient healing, increasing antibiotic use, and costing the global medical sector $386 billion annually (2). Biofilm cells can be up to 1,000 times less susceptible to antibiotics than their planktonic counterparts (3). Exopolysaccharides are a key component of the biofilm matrix, providing protection against host immune responses and antimicrobials (4). These polymers are also involved in anchoring cells to surfaces, facilitating cell-cell interactions, and scaffolding the matrix (5).

One strategy to eliminate biofilms is to inhibit their formation by preventing EPS synthesis. This approach specifically targets the microorganism, as the bacteria will remain in a planktonic state, where they are more susceptible to antimicrobials. Popular approaches to prevent EPS biosynthesis include inhibiting the bacterial secondary messenger bis-(3′–5′)-cyclic diguanylic acid (c-di-GMP) (6, 7) and quorum sensing pathways (8). Despite the critical role that exopolysaccharides play as biofilm matrix components, targeting exopolysaccharide biosynthetic machinery to inhibit production of these key biofilm determinants has been largely overlooked.

Exopolysaccharides are structurally very diverse, with linear and branched homo- and heteropolymers with distinct monosaccharide compositions. These polymers can be further modified post-polymerization through the action of various carbohydrate active enzymes that add or remove functional groups or cleave glycosidic bonds. These modifications impart beneficial characteristics and alter the physicochemical and biological properties of the polymer, which contribute to improved bacterial persistence and survival (9). Common modifications include succinylation, epimerization, acetylation, deacetylation, and glycosidic bond cleavage.

The Pel polysaccharide is a positively charged, partially deacetylated α-1,4-linked *N*-acetylgalactosamine (GalNAc) polymer that is composed predominantly of GalNAc-GalN repeat units (10–12). This polymer appears to be one of the most phylogenetically widespread biofilm matrix determinants, with the Pel biosynthetic loci having been identified in more than 500 diverse Gram-negative and 900 Gram-positive organisms (13, 14). Importantly, deacetylation of the Pel polysaccharide has been shown to be required for biofilm formation in both Pseudomonas aeruginosa and Bacillus cereus ATCC 10987 (13, 15), suggesting that this chemical modification could be a viable target for biofilm prevention across both Gram-negative and Gram-positive organisms.

In P. aeruginosa, the Pel exopolysaccharide is crucial for initiating and maintaining cell-to-cell interactions and providing protection against aminoglycoside antibiotics and mucolytic treatments (11, 16). All seven genes of the P. aeruginosa *pelABCDEFG* operon are necessary for the production of Pel (10–12). Deacetylation of the GalNAc polymer occurs in the periplasm and requires the bifunctional modification enzyme PelA. The N-terminal domain of PelA has glycoside hydrolase activity (17, 18) and is responsible for generating low-molecular-weight, matrix-associated Pel, also referred to in the literature as secreted or cell-free Pel. Matrix-associated Pel contributes to virulence and various biomechanical properties of the biofilm, including pellicle cohesiveness, complex colony wrinkling, matrix hydrophilicity, and stiffness (19). The C-terminal region of PelA contains a metal-dependent deacetylase domain, which must be catalytically active for the formation of Pel-dependent biofilms (15, 20). Whether fully acetylated Pel exopolysaccharide is exported has not been resolved (15).

Herein, we demonstrate that Pel is not found in the extracellular milieu of a P. aeruginosa PelA deacetylase mutant, reinforcing the hypothesis that inhibiting PelA deacetylase activity could be a valid approach to prevent biofilm formation (15). We developed and performed a high-throughput screen (HTS) to identify small molecule inhibitors of PelA’s esterase activity, the first enzymatic step in the deacetylation process. Analyses of biofilm inhibition, structure-activity relationships (SAR), cytotoxicity, and Michaelis-Menten kinetics led to the identification of the first noncompetitive inhibitor of PelA, SK-017154-O, and a potent noncytotoxic Pel-dependent biofilm inhibitor, compound 1, that does not directly inhibit PelA esterase activity. SK-017154-O and its phenyl derivative, compound 1, also inhibit Bacillus cereus Pel-dependent biofilm formation. These results provide a proof of concept that exopolysaccharide modification enzymes can be targeted with small molecule inhibitors to block Pel-dependent biofilm development in both Gram-negative and Gram-positive species.

## RESULTS

### Extracellular Pel is not produced by a PelA deacetylase mutant.

**FIG 1:**
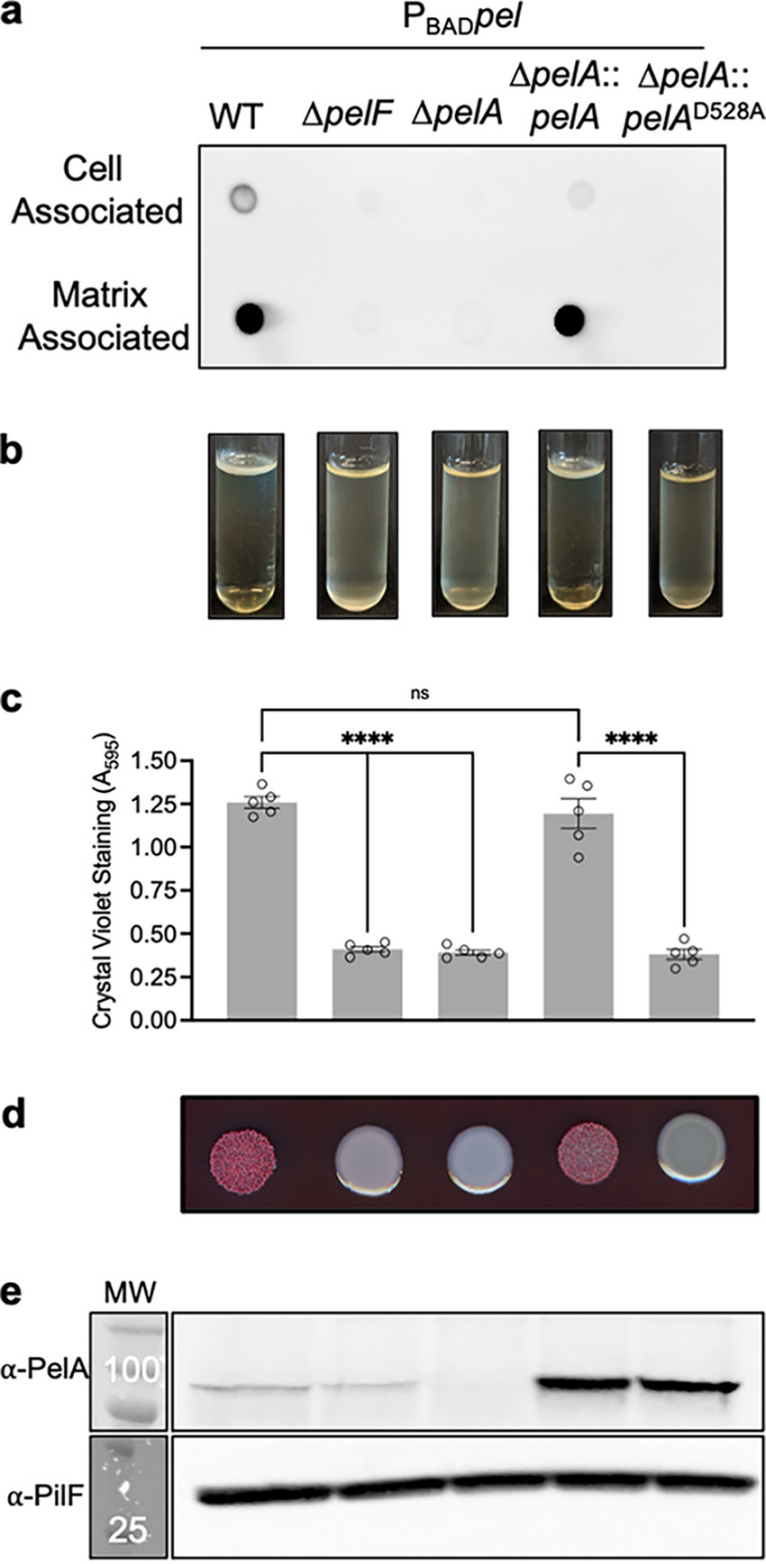
Extracellular Pel is not produced by a PelA deacetylase mutant. (a) Dot blot of crude cell-associated and matrix-associated fractions from indicated strains. Pel was detected using WFL-HRP. (b) Standing pellicle assay; (c) crystal violet microtiter plate assay. Error bars represent the standard error of the mean from five independent trials. Statistical significance was evaluated using an ordinary one-way analysis of variance with Tukey corrections for multiple comparisons. ns, no significant difference; ****, *P* < 0.0001. (d) Congo red colony morphologies; (e) Western blot for PelA and PilF loading control. The molecular weight (MW) is indicated in kilodaltons in the MW marker lane on top of the marker band. The molecular weights of proteins are as follows: PelA, 101.1 kDa; PilF, 28.5 kDa. P_BAD_*pel*, PAO1 Δ*wspF* Δ*psl* P_BAD_*pel*.

PelA deacetylase activity is required for Pel-dependent biofilm formation (15). However, the mechanism by which biofilm formation is abrogated is unknown. Examination of the biosynthesis of poly-β-1,4-*N*-acetylglucosamine (PNAG) in Gram-negative organisms, where disruption of a deacetylase also abolishes biofilm formation, suggests two potential mechanisms. Catalytically inactive mutants of the deacetylase PgaB in Escherichia coli and HmsF in Yersinia pestis do not export PNAG, while deletion of BpsB in Bordetella bronchiseptica results in acetylated PNAG being exported and shed from the cell surface (21–24). In Pel biosynthesis and export, PelC is proposed to act as a dodecameric electronegative funnel that guides deacetylated cationic Pel toward the PelB porin for export into the extracellular milieu. This hypothesis suggests that deacetylation is necessary for export (25). To investigate this hypothesis, we prepared crude cell-associated and matrix-associated fractions from a strain engineered to overproduce Pel (PAO1 Δ*wspF* Δ*psl* P_BAD_*pel*, henceforth called P_BAD_*pel*) (10). Using Wisteria floribunda lectin conjugated to horseradish peroxidase (WFL-HRP), which recognizes terminal GalNAc (26), we probed for the presence of Pel or acetylated Pel (26) (Fig. 1a). The parental P_BAD_*pel* strain served as a positive control, producing both cell- and matrix-associated Pel. The negative-control strains, P_BAD_*pel* Δ*pelF*, in which the glycosyltransferase is deleted, and P_BAD_*pel* Δ*pelA*, which does not form biofilms (19), produced no detectable Pel in either fraction. Complementation of P_BAD_*pel* Δ*pelA* with a wild-type copy of *pelA* restored cell- and matrix-associated Pel production comparable to levels in the parental P_BAD_*pel* strain. As these results were consistent with our previously reported results (19), we next complemented P_BAD_*pel* Δ*pelA* with a *pelA* deacetylase D528A catalytic point mutant variant (15). No cell- or matrix-associated Pel was detected for P_BAD_*pel* Δ*pelA*::*pelA*^D528A^. These results reveal Pel is not found in the extracellular milieu in a PelA deacetylase mutant.

To confirm that the lack of detectable GalNAc moieties in P_BAD_*pel* Δ*pelA*::*pelA*^D528A^ correlates with loss of biofilm formation, we next assessed the capacity of this strain to organize into pellicles. We observed that P_BAD_*pel* Δ*pelA*::*pelA*^D528A^ did not form a pellicle, but rather a turbid culture like the biofilm-deficient P_BAD_*pel* Δ*pelF* and P_BAD_*pel* Δ*pelA* strains (Fig. 1b). Similarly, crystal violet microtiter plate assays, which quantify adherent biofilm biomass, showed that P_BAD_*pel* Δ*pelA*::*pelA*^D528A^ produced very little crystal violet staining (Fig. 1c). We also examined the colony morphologies of the strains, because many Pel-producing strains exhibit rugosity when grown on agar (27). The P_BAD_*pel* Δ*pelF*, P_BAD_*pel* Δ*pelA*, and P_BAD_Δ*pelA*::*pelA*^D528A^ strains all exhibit smooth colony morphologies, suggesting loss of Pel production (Fig. 1d). To ensure that the results observed were not due to the D528A mutation destabilizing the PelA protein, we performed Western blot analyses (Fig. 1e). These revealed, as seen previously (15), that a deacetylase D528A catalytic point mutation does not affect PelA abundance in the P_BAD_*pel* strain background. Collectively, these results suggest that PelA deacetylase activity is required for export of the Pel polymer and that it is essential for Pel-dependent biofilm formation.

### High-throughput screening identified 56 hits that inhibit PelA_Δ46_ esterase activity.

The phenotypes we observed for P_BAD_Δ*pelA*::*pelA*^D528A^ (Fig. 1) validate the deacetylase activity of PelA as a target for a screening campaign to discover small molecules that inhibit Pel-dependent biofilm formation. Deacetylases often have significant esterase activity with pseudosubstrates, which can be used to monitor enzymatic activity. Using the chromogenic acetyl ester 7-acetoxycoumarin-3-carboxylic acid (ACC) as a pseudosubstrate we developed a fluorometric HTS assay (see Fig. S1 in the supplemental material). ACC has been used previously to monitor the esterase activity of the de-*N*-acetylase PgaB from Escherichia coli (28). A total of 69,360 compounds from the ChemBridge, Maybridge, Prestwick, Microsource SPECTRUM, and Library of Pharmacologically Active Compounds (LOPAC) chemical libraries were screened at a final concentration of 40 μM. To evaluate the quality of the ACC assay as a HTS, the Z′ score, a statistical parameter reflective of the signal range and variation of controls, was calculated (29). The average Z′ score across all 217 plates was 0.59 ± 0.05, indicating that the ACC assay has a well-defined hit-window as 1 > Z′ ≥ 0.5 (Fig. S1).

**FIG 2:**
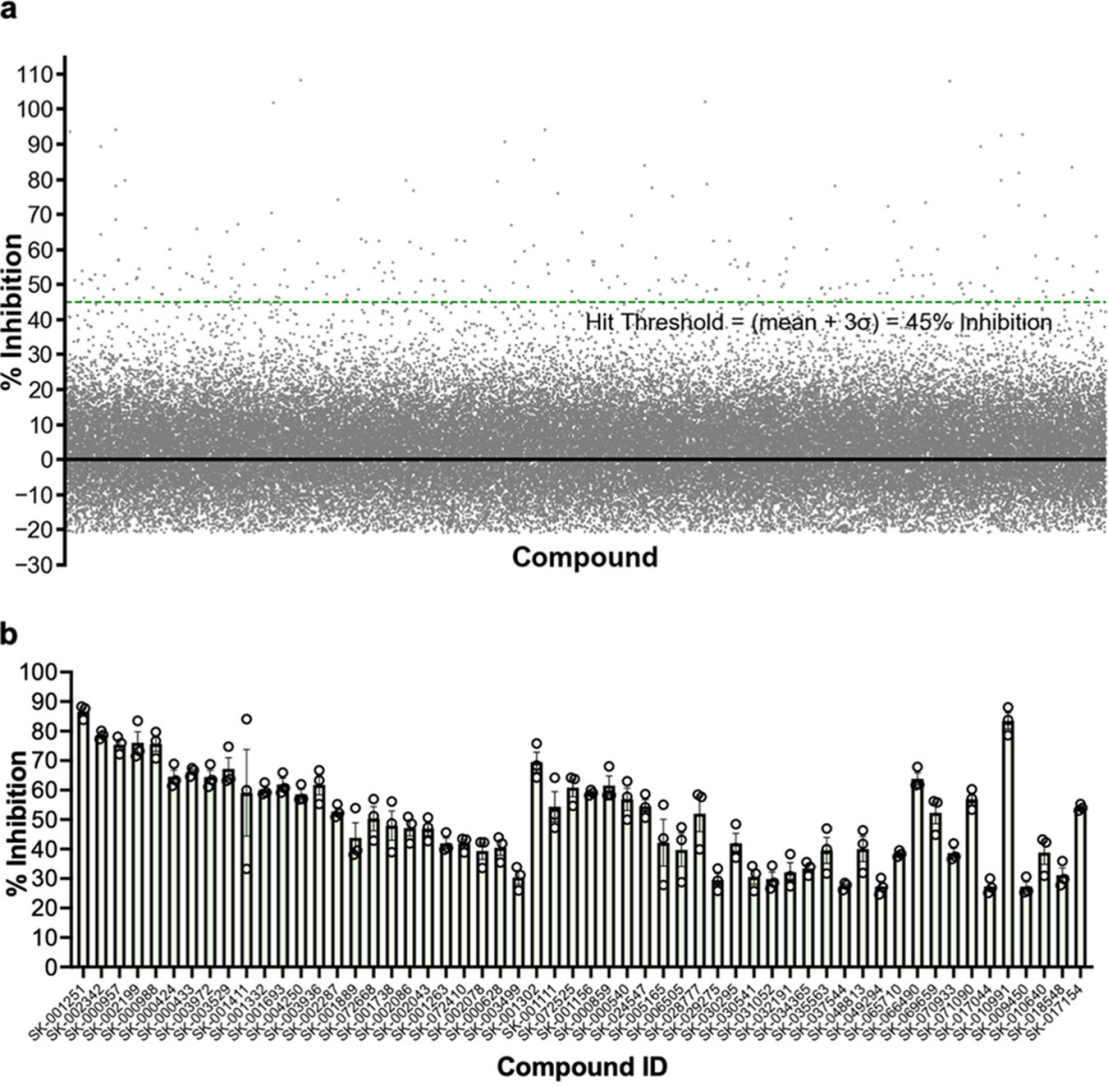
High-throughput screening identified 56 validated hits inhibiting PelA esterase activity *in vitro* (a). High-throughput biochemical screening data of 68,666 compounds. Each point represents inhibition of PelA esterase activity on the pseudosubstrate, ACC, for a given compound tested at 40 μM. Fluorescent and quenching compounds were omitted. The green dashed line represents the calculated hit threshold (mean + 3σ). (b) Fifty-six validated hits from reconfirmation screen. A validated hit was defined as having ≥25% inhibition in three replicates. Error bars represent the standard error of the mean from three independent trials. ACC, 7-acetoxycoumarin-3-carboxylic acid; σ, standard deviation.

Through comparison to the high (PelA only) and low (1% [wt/vol] sodium dodecyl sulfate) controls, fluorescent (false-negative) and quenching (false-positive) compounds were identified (Fig. S1). As a result, 694 fluorescent and quenching compounds were omitted from further analyses. For the remaining 68,666 compounds, the percentage of inhibition was calculated and plotted (Fig. 2a). The mean inhibition for the HTS was 1.7% inhibition, with a standard deviation (σ) of 14.5% inhibition. The hit threshold was defined as the mean percentage of inhibition + 3σ, which equates to 45% inhibition. Using this threshold cutoff, 246 compounds were defined as hits. This corresponds to an initial hit rate of 0.35%. The initial hits were then subjected to a reconfirmation screen using the same ACC assay. In the reconfirmation screen, a confirmed hit was defined as having ≥25% inhibition in all three replicates performed. Using the threshold of 25% inhibition, the reconfirmation screen identified 56 hit compounds (Fig. 2b). In summary, screening 69,360 compounds from diverse chemical libraries identified 56 hits able to inhibit PelA esterase activity *in vitro* in our robust HTS.

### *In vitro* secondary biofilm assays validated four hit compounds.

As inhibition of PelA esterase activity *in vitro* may not translate into inhibition of Pel-dependent biofilm formation, we next explored whether the 56 hits attenuated Pel biofilm formation using a microplate biofilm assay. The P_BAD_*pel* strain was used in this secondary *in vitro* assay as it forms robust Pel-dependent biofilms. We anticipated three potential outcomes for our hit compounds (Fig. S2). A compound could have either no effect on biofilm formation, bactericidal activity, or cause a dose-dependent attenuation of biofilm development. Quantitatively, an absorbance at 595 nm (*A*_595_), which measures biofilm adherence, of <1 with no titratable response and a lack of turbidity suggests a bactericidal effect. Compounds that had either no effect on biofilm development (i.e., did not result in a dose-response curve) or bactericidal activity were not pursued further. Of the 56 hits, 48 had no effect on P_BAD_*pel* biofilm formation and four compounds, SK-000424, SK-000433, SK-072525, and SK-001263, were found to be bactericidal. Four validated hit compounds, SK-049294, SK-017044, SK-034365, and SK-017154 resulted in titratable inhibition of biofilms produced by the P_BAD_*pel* strain (Fig. S2).

### SK-017154 inhibits Pel-dependent biofilm formation in multiple P. aeruginosa strains.

**FIG 3:**
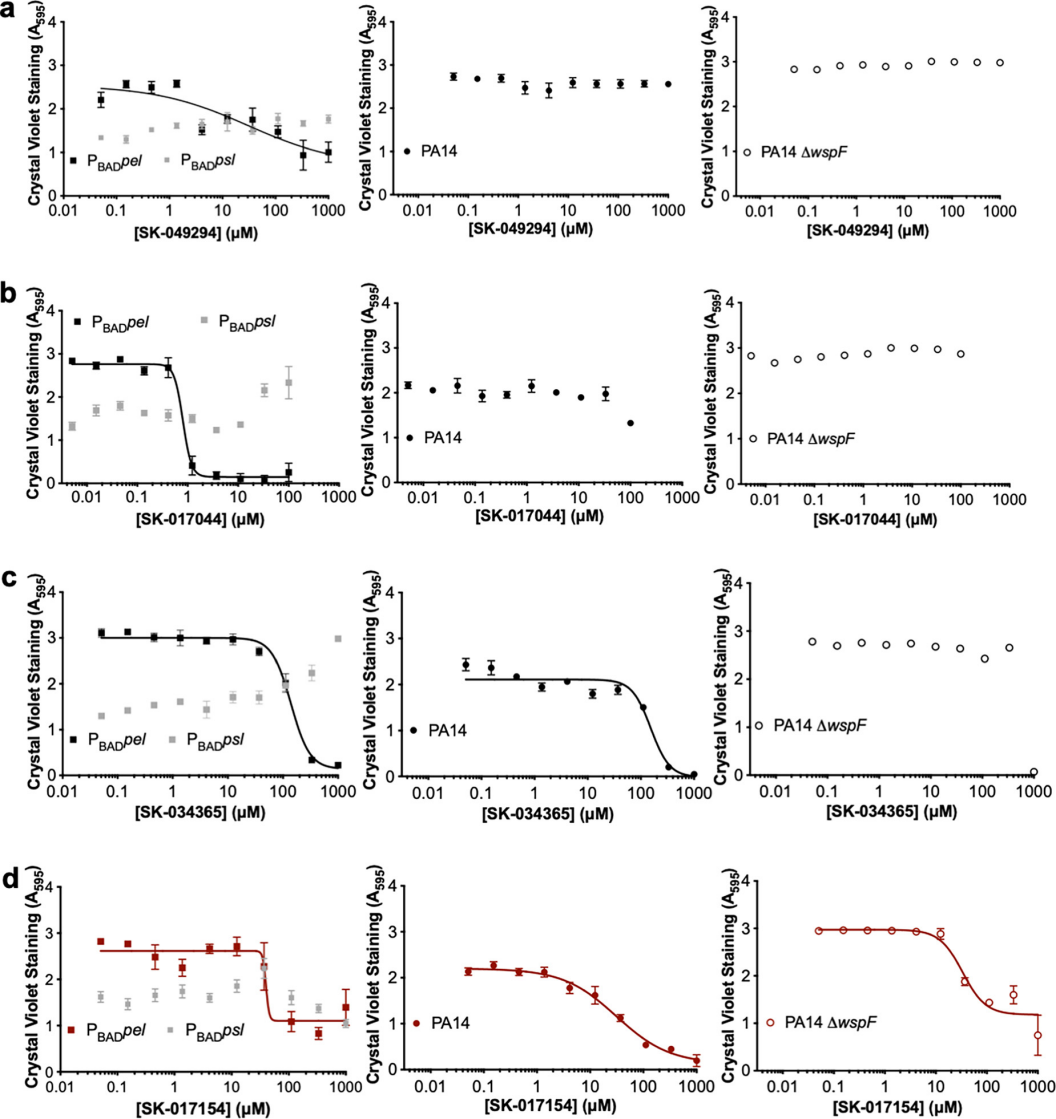
SK-017154 can specifically inhibit P. aeruginosa Pel-dependent biofilm formation in multiple strains. Shown are semilog dose-response curves of the secondary *ex vivo* crystal violet assay using (a) SK-049294, (b) SK-017044, (c) SK-034365, and (d) SK-017154. All validated hits were added to generate 10-point 3-fold serial dilutions. Error bars represent the standard error of the mean from three independent trials. P_BAD_*psl*, PAO1 Δ*wspF* Δ*pel* P_BAD_*psl*; P_BAD_*pel*, PAO1 Δ*wspF* Δ*psl* P_BAD_*pel*.

To prioritize the validated hit compounds for further study, we next examined whether SK-049294, SK-017044, SK-034365, and SK-017154 were able to inhibit biofilm formation in other P. aeruginosa strains. To determine whether the four compounds specifically inhibited Pel-dependent biofilm formation, we next tested their ability to inhibit biofilm formation using (i) an engineered Psl-overproducing strain (PAO1 Δ*wspF* Δ*pelF* P_BAD_*psl*, henceforth called P_BAD_*psl*), (ii) the P_BAD_*pel* strain, (iii) PA14, which produces Pel-dependent biofilms, and (iv) a hyperadherent strain with elevated *pel* expression, PA14 Δ*wspF* (30, 31) (Fig. 3). Only one compound, SK-017154, had dose-dependent inhibition of all three strains producing Pel-dependent biofilms (Fig. 3d). Neither this compound nor the other three compounds tested inhibit biofilms produced by P_BAD_*psl.* SK-017154 was therefore prioritized for further investigation.

### SK-017154 is a 2-pyridinecarboxaldehyde Schiff base of *S*-methyldithiocarbazate.

Prior to further analysis, we sought to verify the structure of SK-017154. ^1^H nuclear magnetic resonance (NMR) analysis of SK-017154 did not generate the expected characteristic singlet for the thiadiazole structure provided by the supplier at ~6 ppm for the thioaminal and a broad singlet around the same region for the N-H (Fig. S3). Instead, a broad singlet at 13.5 ppm and a sharp singlet at 8.25 ppm were observed. The broadness of the singlet at 13.5 suggests that it is the N-H proton. Its presence so far downfield could be rationalized if it is engaged in an intramolecular hydrogen bond with the pyridyl nitrogen (Fig. S3). The presence of the benzylic proton at 8.25 can be rationalized if this carbon is sp2 hybridized. This hypothesis is supported by comparing the spectrum of SK-017154 with one reported for the 2-pyridinecarboxaldehyde Schiff base of *S*-methyldithiocarbazate (32). We synthesized this compound, referred to herein as SK-017154-O, using the protocol provided by Mirza et al. (32) and found its ^1^H NMR spectrum was identical to that of the commercially available compound (Fig. S3). Using SK-017154-O, we repeated the secondary *in vitro* crystal violet assay and found that it robustly inhibited PA14 biofilms with a 50% inhibitory concentration (IC_50_) of 1.8 ± 1.1 μM (Fig. S3). Having established the structure of the compound provided by the supplier and demonstrated that in-house synthesis of this compound produced an inhibitor of PA14 biofilms, SK-017154-O was prioritized for further studies.

### Structure-activity relationship studies reveal pharmacophoric elements for inhibition of biofilms produced by PA14.

**FIG 4:**
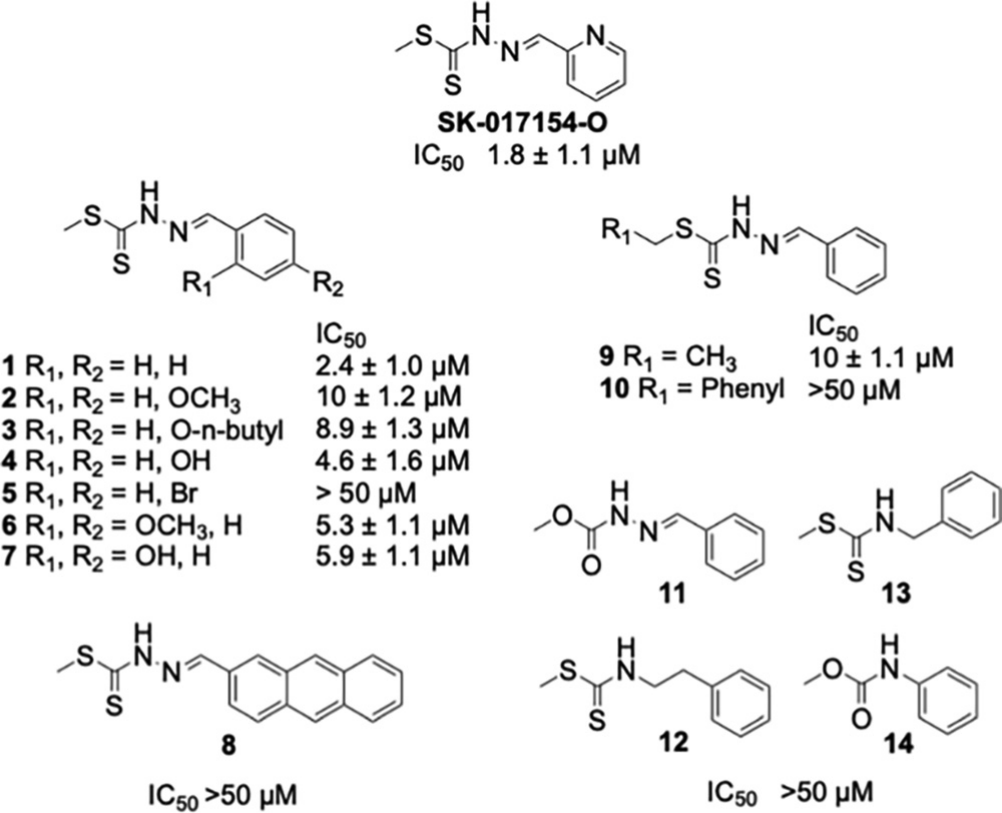
Structure-activity relationship studies reveal pharmacophoric elements for PA14 Pel-dependent biofilm inhibition. IC_50_ values are from six independent trials using the *in vitro* crystal violet biofilm inhibition assay.

To identify the essential elements in the structure of SK-017154-O associated with Pel-dependent biofilm inhibition, we investigated a series of derivatives (Fig. 4). Initially we were interested in substitution of the pyridyl ring of SK-017154-O as pyridyl thiocarbazols are known to be potent metal chelators (32). Synthesis, *via* a known route gave the phenyl derivative, compound 1 (32). Compound 1 had similar biofilm inhibition potency to the parent SK-017154-O, suggesting that pyridyl-directed metal chelation was not an important mechanism in biofilm inhibition by this compound. Further substitution of the phenyl ring (compounds 2 to 8) revealed that ortho- and para-substitution with ether or hydroxy substituents was well tolerated, with moderate losses in binding affinity. Addition of a *p*-bromo substituent to compound 1 or a larger anthracenyl group (compound 8) was not tolerated. Next, we examined whether larger groups could be tolerated in the dithiocarbazate substituent (compounds 9 and 10). Increasing from methyl (compound 1) to ethyl (compound 9) gave a moderate decrease in potency and all inhibition was lost moving to a larger benzyl derivative (compound 10). Finally, we examined variations in the dithiocarbazate linkage. Moving from a dithiocarbazate to carbazate (compound 11), forming sterically similar thiocarbamates (compounds 12 and 13) to compound 1, or moving to a phenyl carbamate (compound 14) resulted in loss of biofilm inhibition potency. From these structure-activity studies, it is evident that the pyridyl nitrogen of SK-017154-O is not required for antibiofilm activity. However, the dithiocarbazate is essential, suggesting a key role in the inhibition of biofilm formation as conservative substitutions on either side of the dithiocarbazate are well tolerated. Given the potency and the expected decrease in metal binding affinity, further biochemical characterization of SK-017154-O and its phenyl derivative compound 1 was carried out. We found that both of these compounds were able to inhibit Pel-dependent biofilms formed by Gram-positive Bacillus cereus ATCC 10987 (13) (Fig. S4).

### SK-017154-O and its phenyl derivative do not inhibit PA14 growth at concentrations that inhibit biofilm production.

**FIG 5:**
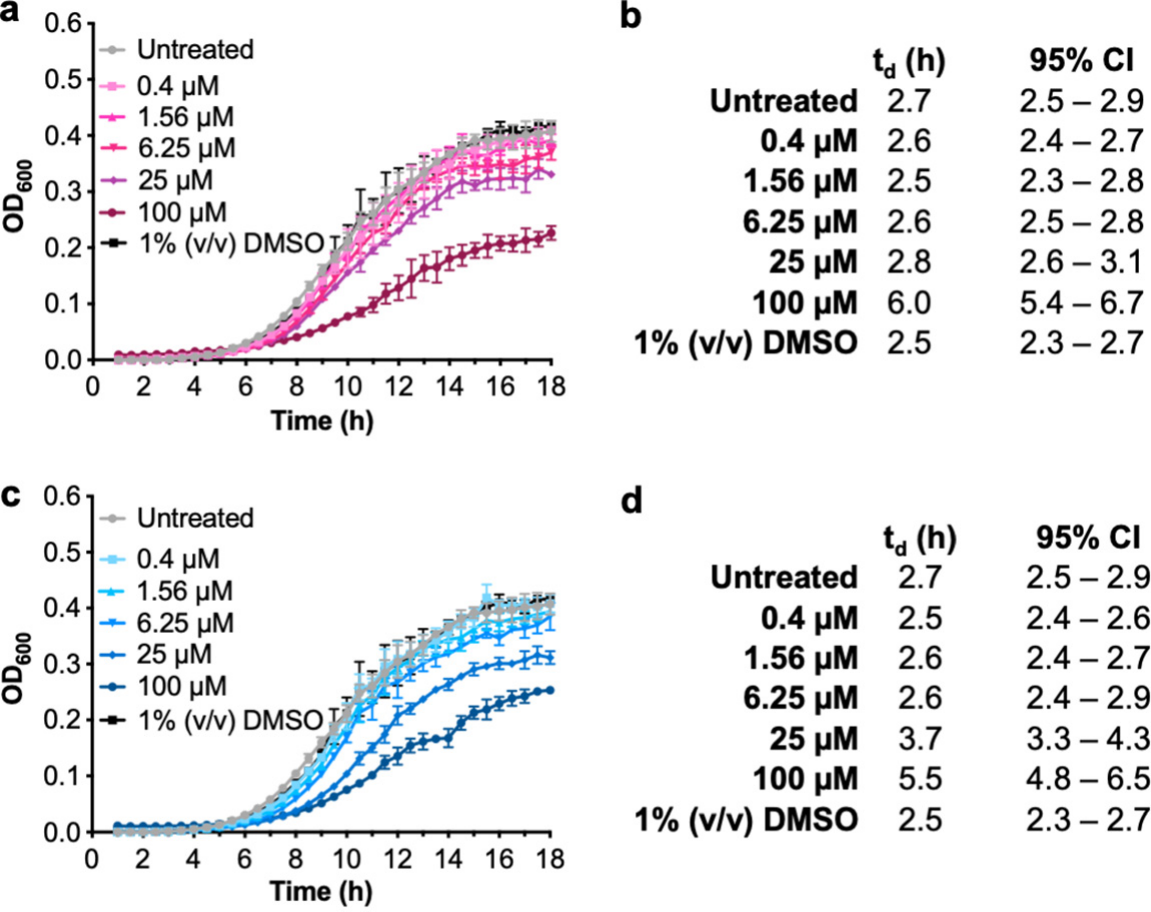
SK-017154-O and compound 1 are not bactericidal to PA14. (a) OD_600_ of planktonic PA14 cells grown with SK-017154-O; (b) calculation of doubling times (*t_d_*) in hours and 95% confidence intervals from panel a; (c) OD_600_ of planktonic PA14 cells grown with compound 1; (d) calculation of *t_d_* in hours and 95% confidence intervals from panel c. Error bars represent the standard error of the mean from three independent trials. DMSO, dimethyl sulfoxide.

To begin to determine the mode of action of SK-017154-O and its phenyl derivative, we first assessed whether these compounds had antimicrobial activity. We examined the growth rates of PA14 with various concentrations of compounds above and below their biofilm IC_50_ values (Fig. 5). Untreated PA14 was used as a positive control, and the addition of 1% (vol/vol) dimethyl sulfoxide (the delivery solvent) to media did not affect the length of the lag or stationary phases or doubling time (33) (Fig. 5). SK-017154-O and compound 1 increased doubling times at concentrations of ≥25 μM (Fig. 5b and d); however, these growth-inhibitory concentrations far exceed the biofilm IC_50_ values of the compounds. This result demonstrates that inhibition of biofilm production is not via bacterial growth inhibition or killing.

### Compound 1 is less cytotoxic to human lung fibroblast cells than SK-017154-O.

**FIG 6:**
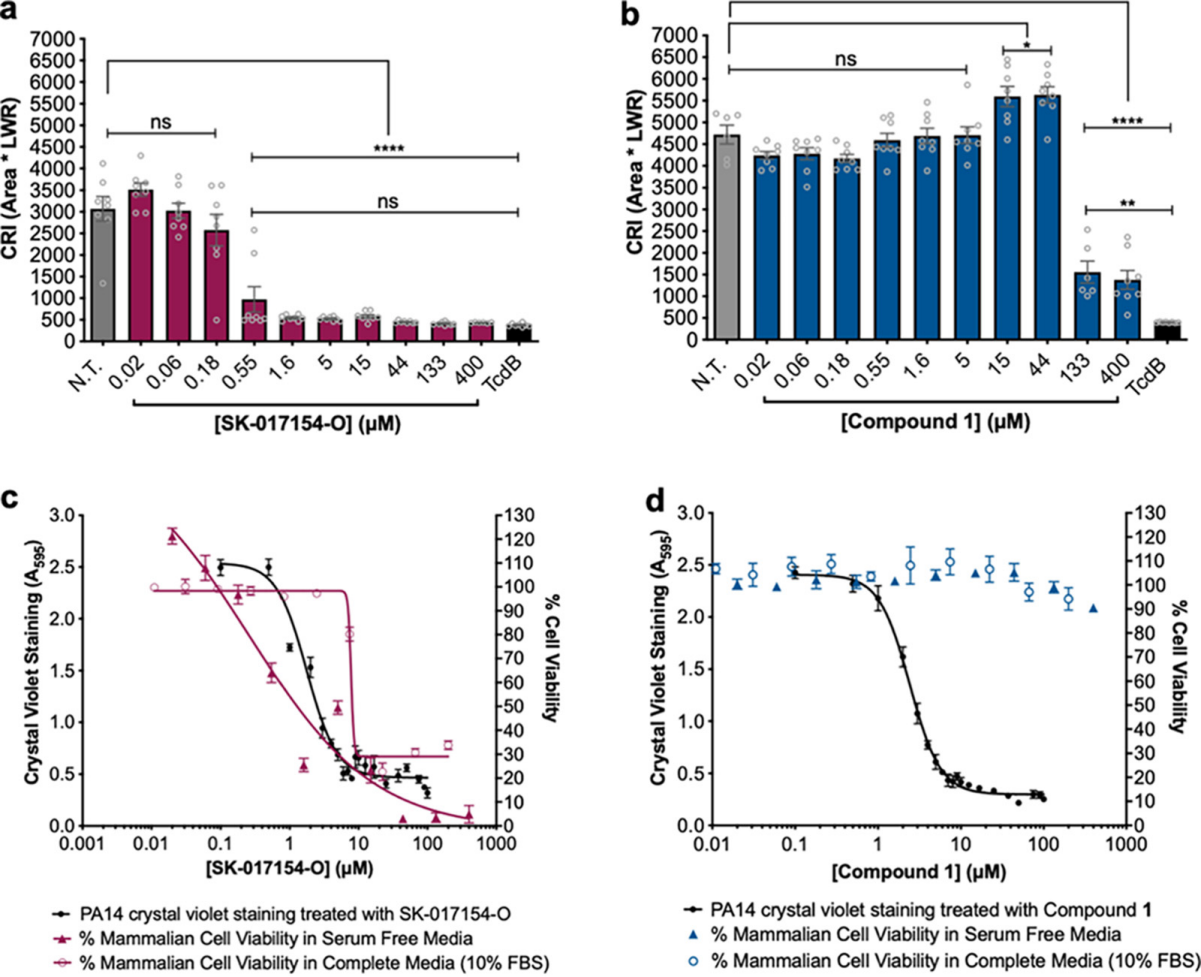
Compound 1 is less cytotoxic to human lung fibroblast cells than SK-17154-O. (a and b) Cell rounding assay against IMR-90 cells using (a) SK-017154-O and (b) compound 1, respectively. Statistical significance was evaluated using an ordinary one-way analysis of variance with Tukey corrections for multiple comparisons. ns, no significant difference (*P* > 0.05); *, *P* ≤ 0.05; **, *P* ≤ 0.01; ****, *P* < 0.0001. Error bars represent standard error of the mean from eight independent trials. (c and d) Cell titer blue cell viability assay against IMR-90 cells using (c) SK-017154-O and (d) compound 1. Error bars represent standard error of the mean from 10 independent trials. CRI, cell rounding index; LWR, length-to-width ratio; FBS, fetal bovine serum.

We next sought to examine whether exogenous compound treatment affected mammalian cell morphology and viability. As cell rounding precedes apoptosis, we monitored changes in cell area (*A*) and the length-to-width ratio (LWR) of IMR-90 human lung fibroblast cells and calculated a cell rounding Index (CRI), where CRI = *A* × LWR. CRI has been used previously to quantify death of a target cell (34). IMR-90 cells treated with ≥0.55 μM SK-017154-O for 24 h had a significantly lower CRI than untreated IMR-90 cells and were not significantly different from the IMR-90 cells treated with the Clostridium difficile toxin TcdB, a positive control for cell rounding (34) (Fig. 6a). This suggests that SK-017154-O influences mammalian cell morphology. Unexpectedly, compound 1 did not influence cell morphology to the same extent, as the treated cells had a significantly higher CRI than TcdB-treated cells even at 400 μM (Fig. 6b). Examination of cellular viability of IMR-90 cells treated with SK-017154-O for 24 h revealed a concentration dependent decrease in viability, regardless of the media used (Fig. 6c). In comparison, compound 1 did not compromise IMR-90 cellular viability even at concentrations where PA14 biofilm development is inhibited, suggesting it has a window between efficacy and toxicity (Fig. 6d). Combined, these results suggest that compound 1 is less cytotoxic than SK-017154-O.

### SK-017154-O inhibits PelA esterase activity *in vitro*.

For enzyme assays with sufficient resolution to determine the mode of inhibition, it is necessary to have active protein and an appropriate *in vitro* assay. When PelAΔ46_Nhis6_ is purified, it was found to be ~85 to 90% pure (15). To improve the purity, we recombinantly expressed and purified PelAΔ46_Chis6_. Not only was PelAΔ46_Chis6_ more homogenous, but it also had a higher specific activity than PelAΔ46_Nhis6_ in an ACC assay (Fig. S5). PelAΔ46_Chis6_ was therefore used in all subsequent assays to determine the inhibitory mode of action of SK-017154-O and its phenyl derivative, compound 1. We first tried to use the pseudosubstrate ACC to kinetically characterize PelAΔ46_Chis6_. However, we found that this compound was prone to autohydrolysis and that saturation of enzymatic activity could not be achieved due to its solubility limit (35) (Fig. S5).

**FIG 7:**
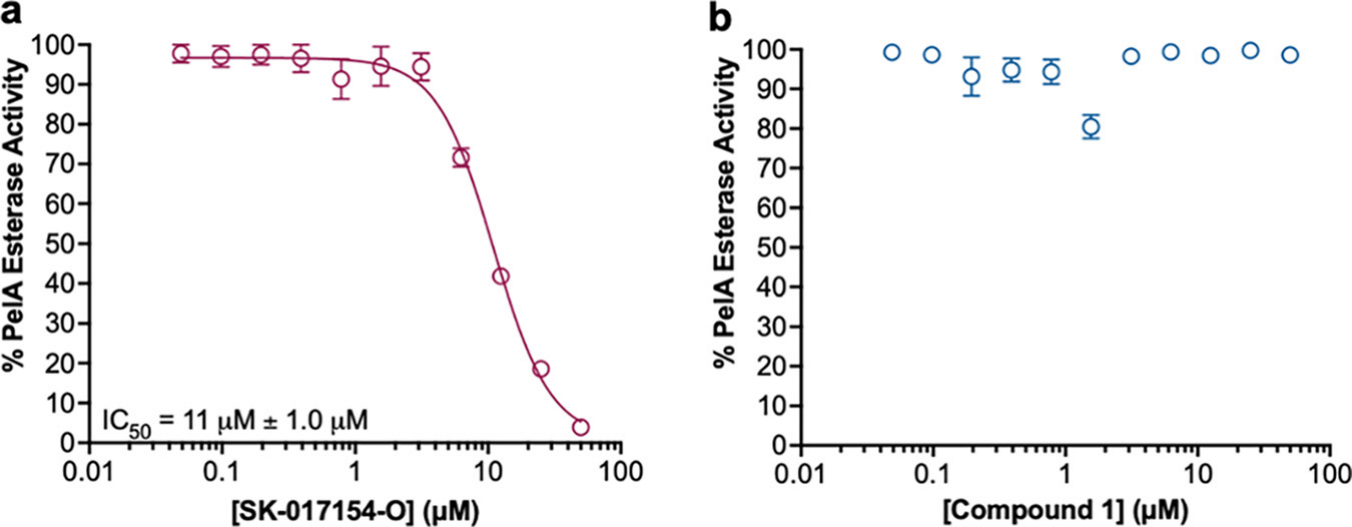
SK-017154-O inhibits PelA esterase activity *in vitro.* AMMU assay results using (a) SK-17154-O and (b) compound 1. Assay mixtures contained 1 μM PelA and 400 μM AMMU. Error bars represent the standard error of the mean from four independent trials. AMMU, acetoxymethyl-4-umbelliferone.

An alternative chromogenic acetyl ester, acetoxymethyl-4-umbelliferone (AMMU), was therefore used as a pseudosubstrate to test the mode of inhibitory action of SK-017154-O and compound 1. AMMU has significantly reduced levels of autohydrolysis and has been used as a suitable pseudosubstrate to characterize the activity of the exopolysaccharide deacetylases PgaB from E. coli and PgdA from Streptococcus pneumoniae (36). Using AMMU, we first sought to generate semilog dose-response plots using PelAΔ46_Chis6_ to determine if SK-017154-O and compound 1 specifically inhibit PelA esterase activity. Analysis of SK-017154-O against PelAΔ46_Chis6_ revealed concentration-dependent inhibition, indicating that the compound specifically inhibits PelA esterase activity (IC_50_ = 11 ± 1.0 μM) (Fig. 7a). Unexpectedly, compound 1 did not inhibit PelA esterase activity (Fig. 7b), despite being a potent inhibitor of PA14 biofilm formation, suggesting it targets something else effecting Pel synthesis. Combined, these results suggest that SK-017154-O may be acting on target, by inhibiting PelA esterase activity, and that compound 1 inhibits Pel-dependent biofilms through an alternative mechanism.

### Michaelis-Menten analyses indicate SK-017154-O is a noncompetitive PelA inhibitor.

**FIG 8:**
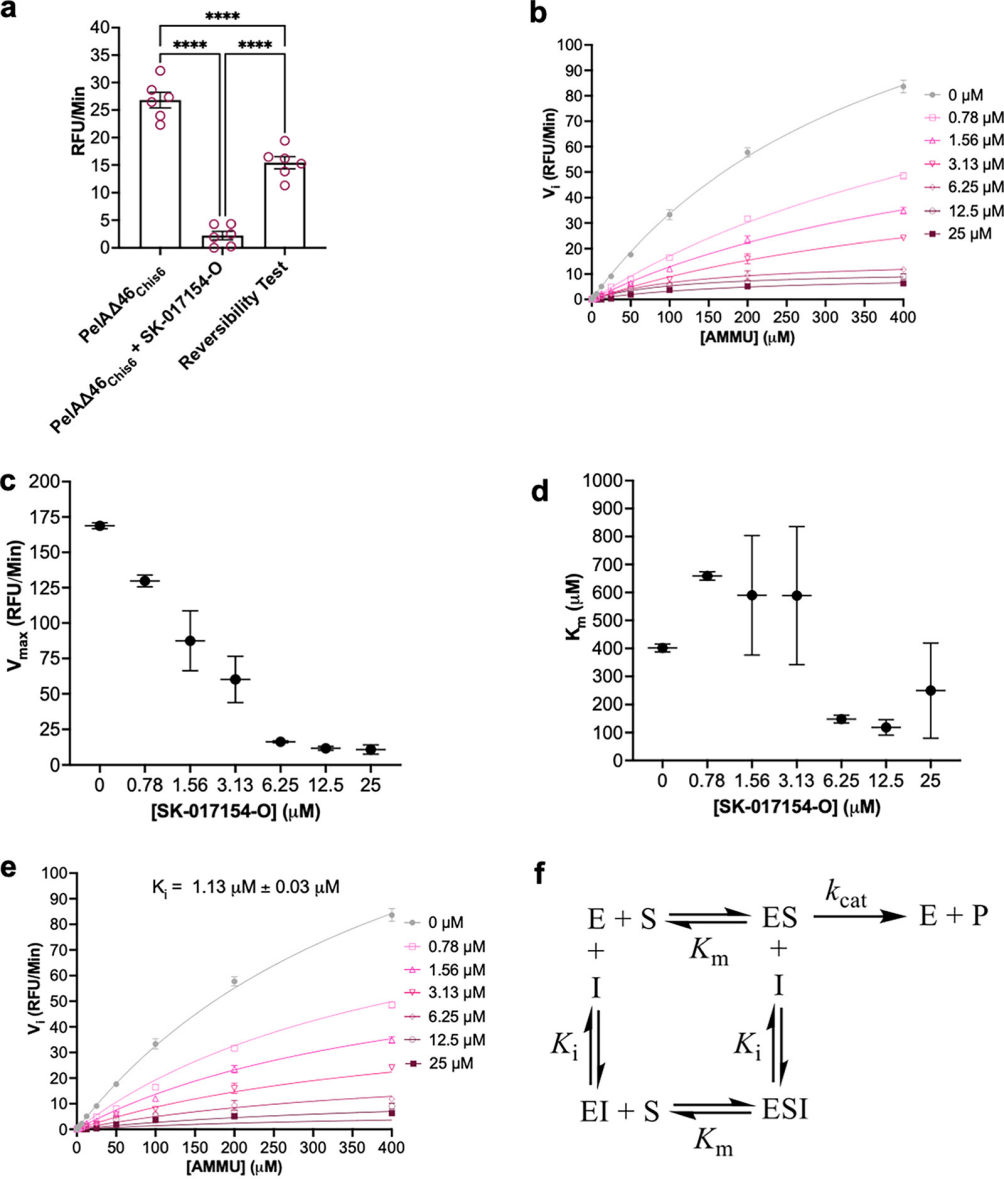
SK-17154-O is a noncompetitive inhibitor. (a) Reversibility test using gel filtration testing SK-017154-O. Assay mixtures contained 1 μM PelA, 25 μM compound, and 400 μM AMMU. Error bars represent the standard error of the mean from six independent trials. Statistical significance was evaluated using an ordinary one-way analysis of variance with Tukey corrections for multiple comparisons. (b) Inhibition kinetics by SK-017154-O generally fit to the Michaelis-Menten equation. Assay mixtures contained 1 μM PelA. (c and d) Apparent *V*_max_ (c) and apparent *K_m_* (d) as a function of SK-017154-O concentration; (e) data in panel a fit to a global noncompetitive inhibition model. Error bars represent the standard deviation from two independent trials. (f) Kinetic scheme of noncompetitive inhibition. RFU, residual fluorescence units; AMMU, acetoxymethyl-4-umbelliferone.

The requirement of the dithiocarbazate for biofilm inhibition and the potential of these compounds to serve as acylating species, albeit at elevated temperature, spurred us to investigate the reversibility of the enzyme inhibition with SK-017154-O (37). Using gel filtration chromatography, enzyme-equilibrated SK-017154-O was separated from the inhibitor and assayed for esterase activity (Fig. 8a). Although the activity of the recovered enzyme was ~60% of that of an uninhibited control, the recovered activity was significantly above that of the enzyme in the presence of the inhibitor, suggesting the inhibition with SK-017154-O is reversible and that steady-state approximations can be used in our Michaelis-Menten kinetics analysis using AMMU.

A decrease in the initial esterase velocities with increasing concentrations of SK-017154-O (Fig. 8b) was observed, consistent with enzyme inhibition. A Lineweaver-Burk plot of the SK-017154-O kinetics revealed high error at lower substrate concentrations, which did not allow the mode of inhibition to be unambiguously determined (Fig. S6). Analysis of the effect of increasing concentrations of SK-017154-O on steady-state kinetic parameters revealed a robust decrease in the apparent *V*_max_ (Fig. 8c) and an ambiguous trend with the apparent *K_m_* (Fig. 8d). To distinguish between noncompetitive and mixed inhibitor modalities, we performed an extra sum of squares *F* test and compared the fit of both models. The mixed inhibition model did not provide sufficient goodness of fit to justify its higher complexity (*P* = 0.11), so the simpler noncompetitive inhibitor modality was chosen. By globally fitting the data to a noncompetitive inhibitor model, we determined the inhibition constant as *K_i_* = 1.13 ± 0.03 μM (Fig. 8e). In summary, our data analysis supports that SK-017154-O is a noncompetitive, reversible PelA deacetylase inhibitor with affinity for both free enzyme and enzyme-substrate complex and that the compound binds to a site distinct from the deacetylase active site (35) (Fig. 8f).

## DISCUSSION

PelA deacetylase activity is necessary for Pel-dependent biofilm formation (13, 15). Expanding on these studies, we found herein that PelA deacetylase activity is necessary for production of extracellular Pel, thus validating the enzyme as a target to inhibit Pel-dependent biofilm formation. Using a HTS approach, we identified the first known noncompetitive inhibitor of PelA esterase activity, SK-017154-O, and revealed using SAR that the dithiocarbazate linkage is the key structural element and that the pyridyl ring can be substituted with phenyl. SK-017154-O and its phenyl derivative, compound 1, inhibit both P. aeruginosa and B. cereus ATCC 10987 Pel-dependent biofilm formation. Our results demonstrate the validity of targeting an exopolysaccharide modification enzyme to prevent biofilm formation.

Mutations that compromise the deacetylase activity of PelA in both Gram-negative P. aeruginosa and Gram-positive B. cereus ATCC 10987 have been shown to attenuate biofilm formation (13, 15). In this work, using dot blot analyses we determined that a P. aeruginosa strain with a point mutation in the catalytic site of the PelA deacetylase domain results in an absence of Pel in the extracellular milieu (Fig. 1a). This is consistent with PNAG production in E. coli and Y. pestis, where deacetylation of the polysaccharide is required for export (21, 22). This finding also supports previous suggestions that Pel deacetylation is important for export (15, 25). The biosynthesis of Pel requires the inner membrane complex of PelD, PelE, PelF, and PelG proteins (38). Once in the periplasm, deacetylation by PelA confers a cationic charge to the polymer. PelA interacts with the tetratricopeptide repeat domain of the outer membrane protein PelB, an interaction that increases the deacetylase activity of PelA. After deacetylation, Pel is then proposed to be guided toward the electronegative funnel of PelC, and thereby the porin domain of PelB, for export from the cell (20, 25). While PelA has been shown to interact with PelB, an interaction between the inner and outer membrane machinery has not been demonstrated to date (20). Given that mutation of a deacetylase active site residue of PelA does not perturb expression of the protein (Fig. 1e), we anticipate that polymerization of Pel is unlikely to be disrupted by a single point mutation that abrogates the deacetylase activity of PelA. We therefore hypothesize that the endo-α-1,4-*N*-acetylgalactosaminidase activity of PelA (18) degrades any acetylated Pel that is synthesized and retained in the periplasm. Transmission electron micrographs of deletion mutants of the PNAG deacetylase PgaB immunostained with a PNAG monoclonal antibody revealed that PNAG is retained in the periplasm (21). This is consistent with our hypothesis as deletion of *pgaB* will also remove the glycoside hydrolase activity that this bifunctional enzyme exhibits. Loss of PgaB’s glycoside hydrolase activity would thus prevent degradation of any accumulating polymer. Similarly, in alginate biosynthesis the periplasmic lyase AlgL has been shown to function as a homeostasis enzyme clearing the periplasmic space of accumulated alginate (39). Further studies are needed to understand the effect that compromised PelA deacetylase activity has on Pel biosynthesis in P. aeruginosa and what effects on polymerization are observed in Gram-positive species where deacetylation occurs extracellularly.

One strategy for biofilm prevention is to inhibit the production of EPS. To date, this strategy has mainly been focused on targeting c-di-GMP and/or quorum sensing signaling networks, which are difficult to control (8). Targeting deacetylation of exopolysaccharides as a therapeutic strategy has been proposed previously for PNAG-dependent biofilm formation (4, 15, 23). Monosaccharide-based metal chelators that inhibit the deacetylase activity of PgaB have been synthesized and shown to inhibit esterase activity *in vitro*, but were unable to inhibit biofilm formation (36, 40). In comparison, our unbiased approach using a HTS and subsequent analyses were successful in identifying two Pel-dependent biofilm inhibitors, SK-017154-O and its phenyl derivative, compound 1 (Fig. 3d; see Fig. S4 in the supplemental material). We found that SK-017154-O, the 2-pyridinecarboxaldehyde Schiff base of *S*-methyldithiocarbazate, is a noncompetitive inhibitor of PelA esterase activity, and while it is nonbactericidal under the conditions tested, it is cytotoxic to IMR-90 cells (Fig. 5, 6, and 8). SK-017154-O is an analog of a potent experimental anticancer drug, triapine (32, 41). Triapine functions as an inhibitor of ribonucleotide reductase, an enzyme found in all living cells that catalyzes the formation of deoxyribonucleotides from ribonucleotides using a free radical mechanism (42–44). Triapine is proposed to inhibit class I ribonucleotide reductases by binding to the surface of the protein, causing release of iron, which leads to catalytic inactivation (41, 45). Triapine can also act as an iron chelator, forming iron(II)-triapine, which can activate O_2_ and in a Fenton-like reaction produce reactive oxygen species (ROS) that could then damage the cell (41). X-ray crystal structures of SK-017154-O with zinc(II) and cadmium(II) reveal that it is an *NNS* tridentate chelating agent that coordinates metal ions in a deprotonated thiolate form *via* the pyridine nitrogen, the azomethine nitrogen, and thiolate sulfur atoms (32). While this suggests that SK-017154-O could be inhibiting PelA’s metal-dependent esterase activity by chelating or rendering labile the active site metal, we found that the inhibition was reversible (Fig. 8a). Structural studies of a PelA:SK-017154-O complex will be needed to determine where this compound binds allosterically to the enzyme and how it inhibits esterase activity. These studies would also allow for development of second-generation inhibitors.

While we and others have not tested SK-017154-O as an inhibitor of ribonucleotide reductase, Mirza et al. have shown that it is cytotoxic to human lymphoblastic leukemia (HL-60) cells (32). This is consistent with the results presented herein that show SK-017154-O is cytotoxic to IMR-90 cells (Fig. 6a and c). The only structural difference between SK-017154-O and its phenyl derivative, compound 1, is the absence of the pyridine nitrogen in the latter. Compound 1 would thus to be unable to function as an *NNS* tridentate chelating agent. This structural difference could explain why compound 1 was noncytotoxic to IMR-90 cells (Fig. 6b and d). P. aeruginosa contains a class I ribonucleotide reductase, which has been used previously in a HTS to discover P. aeruginosa selective antibiotics that inhibit growth (46, 47). Both SK-017154-O and compound 1 had moderate bacteriostatic effects at higher concentrations (Fig. 5). The bacteriostatic effect of SK-017154-O could potentially be explained by a reduced pool of deoxynucleoside triphosphates and DNA polymerase activity following ribonucleotide reductase inhibition (42). Further studies are needed to confirm this hypothesis and to identify the target of compound 1 to determine how it inhibits the production of Pel-dependent biofilms.

Pel biosynthetic loci are widespread across eubacteria (13, 48). The ability of SK-017154-O and its phenyl derivative, compound 1, to inhibit both Gram-negative and Gram-positive Pel-dependent biofilms (Fig. 3; see Fig. S4 in the supplemental material) makes them attractive potential tools for detecting and characterizing Pel-dependent biofilm formation in a wide range of both environmental and clinical strains. In summary, our screening campaign successfully identified compounds that inhibit Pel-dependent biofilms and provides proof of concept that targeting an exopolysaccharide modification enzyme to prevent biofilm formation is efficacious.

## MATERIALS AND METHODS

### Bacterial strains, plasmids, microbiological media, and growth conditions.

The bacterial strains and plasmids used in this study are listed in Table S1 and Table S2, respectively, in the supplemental material. E. coli and P. aeruginosa strains were routinely grown at 37°C overnight, and broth cultures were shaken at 200 rpm. Lysogeny broth (LB) contained the following per liter of ultrapure water: 10 g tryptone, 10 g NaCl, and 5 g yeast extract. No-salt lysogeny broth (NSLB) was prepared as LB, but excluded NaCl. Jensen’s medium contained the following per liter of ultrapure water: 5 g NaCl, 2.51 g K_2_HPO_4_, 15.56 g glutamic acid, 2.81 g l-valine, 1.32 g l-phenylalanine, 0.33 g/L MgSO_4_•7H_2_O, 21 mg CaCl_2_•2H_2_O, 1.1 mg FeSO_4_•7H_2_O, 2.4 mg ZnSO_4_•7H_2_O, and 1.25% (wt/vol) d-glucose. Vogel-Bonner minimal medium (VBMM) (49) was prepared as a 10× stock containing 2.0 g MgSO_4_•7H_2_O, 20.0 g citric acid, 100 g K_2_HPO_4_, and 35.0 g NaNH_4_HPO_4_•4H_2_O, which was adjusted to a pH of 7.0 and filter sterilized. VBMM was prepared, as required, by diluting this 10× stock 10-fold with autoclaved ultrapure water and by adding 1.0 mL of 1,000× trace metal solution to it as previously described (50). The 1,000× trace metal solution contained 13.5 × 10^−2^ g FeCl_3_•6H_2_O, 494.8 × 10^−3^ g MnCl_2_•4H_2_O, 3.7 × 10^−3^ g Zn(NO_3_)_3_•6H_2_O, 27.7 × 10^−3^ g CaCl_2_, 7.0 × 10^−3^ g CoSO_4_•7H_2_O, 4.3 × 10^−3^ g CuSO_4_, 6.0 × 10^−3^ g NaMoO_4_•H_2_O, 123.2 × 10^−3^ g MgSO_4_•7H_2_O, and 32.4 × 10^−3^ g NiCl_2_ per 250.0 mL of Milli-Q water, which was filter sterilized and stored at −20°C. To prepare solid medium, 1.5% (wt/vol) agar was added to LB or NSLB or 1.0% (wt/vol) noble agar was added to VBMM. Antibiotics were added to growth medium, where appropriate. For E. coli, 50 μg/mL kanamycin (KAN), 50 μg/mL carbenicillin (CAR), or 10 μg/mL gentamicin (GEN). For P. aeruginosa, 60 μg/mL GEN was used for merodiploid selection, whereas 30 μg/mL GEN was used for mini-Tn*7* mutant selection.

### Chemical libraries.

The ChemBridge, Maybridge, Prestwick, Microsource, and LOPAC chemical libraries were purchased by the SickKids Proteomics, Analytics, Robotics and Chemical Biology Centre (SPARC) BioCentre at SickKids. The 7-acetoxycoumarin-3-carboxylic acid (ACC) (51) used for high-throughput screening and acetoxymethyl-methylumbelliferone (AMMU) (36) used for enzyme kinetics were synthesized as previously described. Compounds 2 to 8, 12, and 14 were purchased from Sigma. Other analogues were synthesized as described below.

### Synthetic procedures and compound characterizations.

Figure S7 shows the ^1^H and ^13^C NMR analyses of synthesized compounds.

**(i) General procedure for the synthesis of *S*-alkyldithiocarbazates.** Potassium hydroxide (2.3 g, 41 mmol) was dissolved in 15 mL of a 14:1 solution of ethanol and water and cooled on ice. Hydrazine hydrate (2 g, 40 mmol) was added slowly. Following this addition, a solution of carbon disulfide (3 g, 39.4 mmol) in 2.5 mL ethanol was added dropwise over a 1-h period, maintaining the temperature below 10°C. The resulting biphasic solution was transferred to a separatory funnel, and the pale-yellow top layer was isolated and diluted into a 10-mL solution of 4:6 ethanol and water and then cooled on ice. Alkyl bromide (1 eq) was added to this solution dropwise with vigorous stirring and left for 10 min. At this point, 10 mL of ice-cold water was added and the reaction mixture was left for a further 10 min. The mixture was then filtered, and the solid was washed with water and subsequently dried under vacuum and used immediately and without any further purification.

Crude *S*-alkyldithiocarbazate (1 eq) was dissolved in a 1:1 solution of ethanol and water, to which was added 1 equivalent of aldehyde. The reaction mixture was left for 10 min before the reaction mixture was filtered, and the solid was washed thoroughly with cold ethanol and dried under vacuum.

Analytical data for 2-pyridinecarboxaldehyde *S*-methyldithiocarbazate (SK17154-O) are as follows. ^1^H NMR (dimethyl sulfoxide [DMSO]-d6, 400 MHz): δ = 13.47 (s, 1H), 8.65 (m, 1 H), 8.29 (s, 1H), 7.97 to 7.87 (m, 2H), 7.47 (m, 1H) 2.56 (s, 1H). ^13^C NMR (DMSO-d6, 100 MHz): δ = 199.3, 152.3, 149.8, 146.5, 137.0, 124.9, 120.0, 16.84. HRMS: [M + H]+ calculated, C8H9N3S2 212.03161; found, 212.03105.

Analytical data for benzaldehyde *S*-methyldithiocarbazate (compound 1) are as follows. ^1^H NMR (DMSO-d6, 500 MHz): δ = 13.29 (s, 1H), 8.23 (s, 1H), 7.70 (m, 2H), 7.44 (m, 3H), 2.51 (s, 3H). 13C NMR (DMSO-d6, 133 MHz): δ = 198.8, 146.8, 133.8, 131.2, 129.4, 127.9, 17.24.

Analytical data for benzaldehyde *S*-ethyldithiocarbazate (compound 9) are as follows. ^1^H NMR (DMSO-d6, 400 MHz): δ = 13.25 (s, 1H), 8.26 (s, 1 H), 7.75 (m, 2H), 7.47 (m, 3H), 3.20 (dd, 2H),1.30 (t, 3H). ^13^C NMR (DMSO-d6, 100 MHz): δ = 197.3, 146.4, 133.4, 130.7, 129.0, 127.4, 27.34, 13.83.

Analytical data for benzaldehyde *S*-benzyldithiocarbazate (compound 10) are as follows. ^1^H NMR (DMSO-d6, 400 MHz): δ = 13.38 (s, 1H), 8.27 (s, 1 H), 7.71 (m, 2H),7.49 to 7.41 (m, 5H), 7.35 (m, 2H), 7.29 (m, 1H), 4.51 (s, 2H). ^13^C NMR (DMSO-d6, 100 MHz): δ = 196.6, 146.6, 136.8, 133.3, 130.8, 129.2, 129.0, 128.5, 127.5, 127.2, 37.60.

**(ii) Synthesis of benzaldehyde methylcarbazate (compound 11).** Dimethylcarbonate (1.8 mL, 21 mmol) and hydrazine hydrate (0.97 mL, 20 mmol) were mixed together and heated at 50°C for 20 min. At this point they were allowed to return to room temperature and left overnight. The crude product was dried under vacuum and used immediately and without any further purification. Analytical data are as follows. ^1^H NMR (DMSO-d6, 400 MHz): δ = 11.11 (br s, 1H), 8.04 (s, 1H), 7.64 (m, 2H), 7.46 to 7.36 (m, 3H), 3.71 (s, 3H). ^13^C NMR (DMSO-d6, 100 MHz): δ = 134.3, 129.5, 129.7, 126.6, 51.39.

Methylcarbazate (500 mg, 2.77 mmol) was dissolved in 12 mL of a 1:1 mixture of toluene and diethyl ether, to which benzaldehyde (0.62 mL, 3.05 mmol) was added. The mixture was left overnight before the reaction mixture was filtered and the solid was washed thoroughly with cold ethanol and dried under vacuum.

**(iii) Synthesis of methyl 2-phenylethyl carbamodithioate (compound 12).** Potassium hydroxide (0.28 g, 5 mmol) was dissolved in 2 mL of a 14:1 solution of ethanol and water and cooled on ice. Phenethylamine (0.63 mL, 5 mmol) was added slowly. Following this addition, a solution of carbon disulfide (0.3 mL, 5 mmol) in 0.5 mL ethanol was added dropwise. The resulting biphasic solution was transferred to a separatory funnel, and the pale-yellow top layer was isolated and diluted into a 1-mL solution of 1:1 ethanol and water and then cooled on ice. Methyl iodide (0.31 mL, 5 mmol) was added to this solution dropwise with vigorous stirring and left for 10 min. At this point, 5 mL of ice-cold water was added and the reaction was left for a further 10 min. The mixture was then filtered, and the solid was washed with water and subsequently dried under vacuum. The compound was purified via column chromatography using dichloromethane/MeOH. Analytical data are as follows. ^1^H NMR (CDCl_3_, 500 MHz): δ = 7.34 (m, 3H), 7.22 (m, 2H), 4.01 (dd, 2H), 2.98 (t, 2H), 2.61 (s, 3H). ^13^C NMR (CDCl_3_, 133 MHz): δ = 138.1, 128.8, 129.7, 126.8, 105.0, 47.96, 34.24, 18.10.

### Standard molecular methods.

All basic microbiological and molecular procedures were executed according to standard protocols (52). Genomic DNA (gDNA) was isolated using Bio-Rad InstaGene matrix. Plasmid isolation and DNA gel extraction were performed using purification kits purchased from Qiagen or BioBasic. Restriction enzymes, T4 DNA ligase, Quick ligase, alkaline phosphatase, and *Taq* DNA polymerase were purchased from New England Biolabs or Thermo Fisher Scientific. Phusion DNA polymerase and BP Clonase II were purchased from Thermo Fisher Scientific. Primers used in this study were obtained from Sigma-Aldrich or Integrated DNA Technologies (Table S3). Site-directed mutagenesis of plasmids was completed using the Agilent QuikChange Lightning site-directed mutagenesis kit. Transformation of P. aeruginosa was performed using standard protocols for electroporation (53). Sanger sequencing to validate the sequence of plasmids and chromosomal mutations (described below) was performed at University Core DNA Services in Calgary or The Center for Applied Genomics (TCAG) at SickKids.

### Construction of mini-Tn*7* vectors and transposon mutagenesis.

A mini-Tn*7* vector encoding PelA^D528A^ was constructed via site-directed mutagenesis of pCAS4 (20) (Table S2), which contains the wild type *pelA* gene fused to *araC*::*P*_BAD_ in the Gateway cloning site of pUC18-miniTn7T2.1-Gm-GW (54). An amino acid substitution in PelA encoded by pCAS4 was introduced using the QuikChange Lightening site-directed mutagenesis kit (Agilient). Briefly, primers (oJDR41 and oJDR42) (Table S3) were designed based on the parameters described by the manufacturer and were used in PCR to clone pCAS4 containing the desired mutation. Subsequently, DpnI was added to digest the template plasmids. This reaction mixture was transformed into E. coli XL10-Gold (Table S1) and then spread on LB agar containing 50 μg/mL CAR and 10 μg/mL GEN. Several colonies were picked, streaked on LB agar containing antibiotic selection, and incubated overnight at 37°C. Subsequently, plasmids were purified from these strains. A plasmid with the desired sequence was identified using Sanger sequencing with the primers oJDR33 to oJDR40 (Table S3). This yielded the plasmid pJDR45, which contains a mutant *pelA* allele (encoding PelA^D528A^) fused to *araC*::*P*_BAD_ in the Gateway cloning site of pUC18-miniTn7T2.1-Gm-GW (Table S2). This mini-Tn*7* transposon was introduced into the chromosome of the target P. aeruginosa JJH879 strain (Table S1) via established methods for coelectroporation with the helper plasmid pTNS2. Insertion of mini-Tn*7* at the neutral attTn7 site adjacent to P. aeruginosa *glmS* was confirmed by PCR using the primers PTn7L, PTn7R, PglmS-up, and PglmS-down (Table S3) as previously described (55).

### Allelic exchange.

In-frame and unmarked *wspF* gene deletions were constructed in P. aeruginosa PAO1 Δ*pelF* P_BAD_*psl* using established protocols for two-step allelic exchange (56). The pEX18Gm::Δ*wspF* allelic exchange plasmid was previously constructed (57) and introduced into PAO1 Δ*pelF* P_BAD_*psl* (58) via biparental mating with the donor strain E. coli SM10 (λ_pir_) (59). Merodiploids were selected on VBMM agar containing 60 μg/mL GEN. Double-crossover mutations were selected for by SacB-mediated counterselection on NSLB agar containing 15% (wt/vol) sucrose. Colonies were then screened for those that were GEN sensitive and sucrose resistant. Primers targeting the outside flanking regions of the *wspF* ORF were used to validate the mutations by colony PCR and Sanger sequencing (Table S3).

### Dot blots.

P_BAD_*pel*-derived strains were grown overnight at 37°C to stationary phase in Jensen’s medium supplemented with 0.5% (wt/vol) l-(+)-arabinose to induce *pel* expression. Crude cell-associated Pel and secreted Pel were analyzed using dot blots developed with WFL conjugated to horseradish peroxidase (WFL-HRP) as described by Whitfield et al. (13).

### Standing pellicle assay.

P_BAD_*pel*-derived strains were grown to stationary phase in NSLB and then diluted to a final optical density at 600 nm (OD_600_) of 0.005 in 3 mL of NSLB in borosilicate glass tubes supplemented with 0.5% (wt/vol) l-(+)-arabinose to induce *pel* expression. The glass tubes were left to grow statically at 25°C for 72 h. The assay was performed in triplicate, with one representative image shown.

### Crystal violet microtiter plate assay.

Overnight cultures of P_BAD_*pel*-derived strains were grown to stationary phase in NSLB, diluted to a final OD_600_ of 0.005 in 1 mL of NSLB, and supplemented with 0.5% (wt/vol) l-(+)-arabinose to induce *pel* expression. One hundred microliters of the normalized cultures was added to the wells of a Corning CellBind 96-well microtiter plate (*n* = 5 for each strain examined) and incubated for 20 h at 25°C statically. Nonadherent biomass was removed by washing the wells three times with water, and the remaining adherent biomass was stained with 150 μL of 0.1% (wt/vol) crystal violet for 10 min with agitation at room temperature. To remove excess stain, the wells were washed three times with water and the residual stain was solubilized by adding 200 μL of anhydrous ethanol to each well and left to incubate for 10 min with agitation at room temperature. Forty microliters of the solubilized crystal violet solution was transferred to a plate containing 160 μL of anhydrous ethanol, and the absorbance was measured at 595 nm to quantify the adherent biofilm biomass. B. cereus ATCC 10987 biofilms were grown, stained, and destained as outlined by Whitfield et al. (13).

### Congo red colony morphologies.

Colony biofilms were grown as previously described by Razvi et al. (19).

### Western blot sample preparation and analysis.

P_BAD_*pel*-derived strains were analyzed for protein levels using an anti-PelA antibody at a dilution of 1:500 (60). Anti-PilF was used a loading control at a dilution of 1:2,000 (61). Samples were prepared and analyzed and the Western blot was imaged as outlined by Razvi et al. (19).

### PelA protein cloning, expression, and purification.

The expression and purification of recombinant N-terminally His_6_-tagged full-length PelA spanning residues 47 to 948 (PelAΔ46_Nhis6_) were performed as described previously (15), and the preparation was divided into aliquots, and stored at −80°C for subsequent use in high-throughput screening.

Vectors for expression of C-terminally His_6_-tagged full-length PelA spanning residues 47 to 948 (PelAΔ46_Chis6_) were generated as follows. The nucleotide sequence of *pelA* from Pseudomonas aeruginosa PAO1 was used to design primers for PCR (Table S3) (62). NdeI and XhoI restriction sites were included in the forward and reverse primers, respectively. The PCR product was cloned into the pET-24a vector following digestion with NdeI and XhoI (Thermo Fisher Scientific). The resulting expression plasmid, pER-PelA^47-948^, was transformed into E. coli Top10 and selected on LB agar containing 50 μg/mL KAN. The plasmids were subsequently purified and then verified by Sanger sequencing using the primers oER7 to oER10. Expression was achieved by transforming pER-PelA^47-948^ into E. coli BL21CodonPlus(DE3)-RP competent cells, growing in 6 L of LB broth supplemented with 50 μg/mL KAN at 37°C to mid-log phase, whereupon protein expression was induced by the addition of isopropyl β-d-1-thiogalactopyranoside (IPTG) to a final concentration of 1 mM. Following induction, the cells were left to grow overnight at 18°C for 20 h, prior to being harvested via centrifugation at 6,000 × *g* for 15 min at 4°C. The resulting cell pellets were stored at −20°C until needed for purification.

PelAΔ46_Chis6_ was purified as follows. Cell pellets from a 6-L bacterial culture were thawed and resuspended in 120 mL of buffer A (20 mM HEPES [pH 8.0], 300 mM NaCl, 20 mM imidazole, and 10% [vol/vol] glycerol). Cells were then lysed by three passes through a homogenizer at 1,000 lb/in^2^ while on ice. The resulting lysate was centrifuged at 30,000 × *g* for 30 min at 4°C to pellet insoluble proteins. The supernatant was run through a Ni^2+^-nitrilotriacetic acid (Ni-NTA) affinity HisTrap HP (GE Healthcare) cartridge using a fast protein liquid chromatography (FPLC) device, washed with buffer A until an *A*_280_ of <100 milli-absorbance units (mAU) was detected, and then eluted with a 10-column volume linear gradient to 100% buffer B (20 mM HEPES [pH 8.0], 300 mM NaCl, 500 mM imidazole, and 10% [vol/vol] glycerol). Samples were taken throughout the purification process and run on a 10% SDS-PAGE gel to assess protein purity. The appropriate elution fractions were concentrated and further purified and buffer exchanged into buffer C (20 mM HEPES [pH 8.0], 150 mM NaCl, and 10% [vol/vol] glycerol) by size exclusion chromatography (SEC) using a HiLoad 16/60 Superdex 200 gel filtration column (GE Healthcare). Samples from SEC were run on a 10% SDS-PAGE gel to verify homogeneity. The protein concentration was determined using the Pierce bicinchoninic acid (BCA) protein assay kit from Thermo Scientific. PelAΔ46_Chis6_ was divided into aliquots and stored at −80°C for subsequent use in enzyme kinetics using AMMU.

### Fluorogenic high-throughput biochemical inhibitor screen using ACC.

The ACC assay was adapted as a HTS and used as a reconfirmation screen as follows. Frozen PelAΔ46_Nhis6_ aliquots were thawed at room temperature for 30 min and diluted with protein buffer (20 mM Tris-HCl [pH 8.0], 150 mM NaCl, 10% [vol/vol] glycerol) to create solution A. Solution A with reaction buffer (50 mM HEPES [pH 7.0] and 75 mM NaCl) was used to prepare high (solution A and reaction buffer at 4°C) and low (solution A and reaction buffer with 2.5% [wt/vol] SDS) control solutions. To a 96-well polypropylene V-bottom plate (Costar; 3363), 225 μL of the low and high control solutions was added to wells in column 1 and columns 2 to 12, respectively, to create a PelA master mix plate. From the PelA master mix plate, 10 μL of the low control solution was added to wells in columns 1 and 2 and 10 μL of the high control solution was added to wells in columns 3 to 24 of a 384-well, black, flat-bottom assay plate (Costar; 3573) (Fig. S1).

Into a 96-well clear V-bottom polypropylene deep well plate (Axygen), 550 μL of reaction buffer was aliquoted and used to transfer 24.5 μL of reaction buffer to a 384-well, clear, round-bottom polypropylene plate (Corning; 3657) to generate a drug dilution plate. From chemical libraries in 384-well plates (Echo), 0.5 μL was diluted in the drug dilution plate such that all compounds were at a concentration of 200 μM and 2% (vol/vol) dimethyl sulfoxide (DMSO).

To begin the HTS assay, 5 μL of compound in the drug dilution plate was added to the wells in columns 3 to 22 of the 384-well assay plate (Fig. S1) and left to preincubate with PelAΔ46_Nhis6_ statically at room temperature for 30 min. To prepare substrate, 1.2 mL of substrate bulk solution (10 mg of ACC and 6.45 mL of DMSO) was mixed with 28.8 mL of reaction buffer to produce the substrate assay solution. To a 96-well polypropylene V-bottom plate (Costar; 3363), 250 μL of substrate assay solution was aliquoted, from which 10 μL was added to the entire assay plate to initiate the reaction. Using an EnVision plate reader, the residual fluorescence units (RFU) of each assay plate were measured immediately after addition of substrate (*T* = 0) and after 3 h of incubation at room temperature using an λ_em_ of 355 nm and λ_ex_ of 460 nm. All liquid handling between plates was performed with an Agilent Bravo liquid handler using 30-μL tips. All reaction mixtures had final concentrations of 0.3 μM PelAΔ46_Nhis6_, 100 μM ACC, and 2% (vol/vol) DMSO. All drug-treated reaction mixtures had 40 μM compound from a chemical library.

The baseline read at *T* = 0 was used to discern which compounds were fluorescent (RFU greater than 2× the average RFU of the high control wells) and which compounds were quenchers (RFU less than half of average RFU of the low control wells). A total of 694 compounds (1% of all compounds screened) were flagged as fluorescent or quenching. These compounds were not included in percentage of inhibition, mean, and standard deviation calculations. The percentage of inhibition was calculated with the measured RFU values as follows:

$$\%\text{ inhibition}=1-\left(\frac{\text{drug-treated well}}{\text{high control avg}}\right)\times 100$$

The Z′ value was calculated for each assay plate using the following equation, where σ is the standard deviation, μ is the mean, H indicates the high control, and L indicates the low control:

$$Z^{\prime }=1-\left(\frac{3\sigma_{\text{(H)}}+3\sigma_{\text{(L)}}}{\mu_{\text{(H)}}-\mu_{\text{(L)}}}\right)$$

The HTS data were plotted using the Python matplotlib.pyplot subpackage on Jupyter Notebooks. Prism (GraphPad Software, Inc.) was used to plot the reconfirmation screen data.

### Secondary *in vitro* biofilm inhibition assay.

Overnight cultures of P. aeruginosa grown to stationary phase in NSLB were diluted to a final OD_600_ of 0.005 in 1 mL of NSLB, where P_BAD_*pel* was supplemented with 0.5% (wt/vol) l-(+)-arabinose to induce *pel* expression. Into 96-well polystyrene microtiter plates (Nunc), 99 μL of normalized culture and 1 μL of hit compound solution dissolved in DMSO were aliquoted. Hit compounds were added in duplicate or triplicate to generate 6- to 10-point dose-response curves in 2-fold or 3-fold dilutions starting at 1 mM or below depending on the solubility of the compound (1% [vol/vol] DMSO final concentration in all wells). Assays after assessing validation compounds were performed as six independent experiments (*n* = 3 wells per compound concentration per experiment). Plates were incubated statically for 24 h at 25°C. Bacterial cultures were visually examined for a lack of turbidity to identify bactericidal activity. Bactericidal hit compounds were excluded from further analyses. Nonadherent biomass was removed and stained with 0.1% (wt/vol) crystal violet as outlined above for the crystal violet microtiter plate assay. The absorbance was measured at 595 nm to quantify the adherent biofilm biomass. Half-maximal inhibitory concentrations (IC_50_) were calculated using nonlinear least-squares fitting to a dose-response model in Prism (GraphPad software). The IC_50_ was the concentration of hit compound required to reduce the adherent biofilm biomass by half after 24 h of treatment.

### Growth rate measurements and doubling time calculations.

Overnight cultures of P. aeruginosa PA14 grown to stationary phase in NSLB were diluted to a final OD_600_ of 0.05 in 1 mL of NSLB. Growth curves were set up and read in 96-well polystyrene microplates (Nunc). Wells treated with compound contained 99 μL of normalized culture and 1 μL of compound solution dissolved in DMSO. Solvent controls contained, 99 μL of normalized culture and 1 μL of DMSO. Untreated controls contained 100 μL of normalized culture. All compounds and controls were tested in triplicate. Plates were sealed with HDClear packaging tape (Duck) and placed inside a Synergy Neo2 (BioTek) plate reader at 25°C with continuous orbital shaking for 18 h, and the OD_600_ was read every 30 min. Growth rate (*r*) was estimated as follows, using the exponential growth formula for continuous growth (63):

$$r=\frac{\ln({\text{OD}}_{t}/{\text{OD}}_{0})}{t}$$

ln is the natural logarithm, OD*_t_* is OD_600_ at time *t*, and OD_0_ is the optical density at the initial time. Corresponding to this equation, *r* was calculated using linear regression of ln(OD_600_) values plotted against time in Prism v.7.05 (GraphPad Software), thereby providing 95% confidence intervals for *r*. The coefficient of correlation (*r*^2^, or goodness of fit) was used to guide selection of datum points from exponential growth that best fit the linear relationship. Subsequently, doubling time, *t_d_*, was calculated as follows:

$$t_{d}=\frac{\ln(2)}{r}$$

The 95% confidence intervals for *t_d_* were calculated from the corresponding *r* values from linear regression.

### Cell rounding assay.

Using freshly dissolved compound in DMSO, the cell rounding assay was performed and a cell rounding index (CRI) was calculated as outlined for the ArrayScan high-content phenotypic screen by Tam and Beilhartz et al. (34), where hit compounds were incubated with IMR-90 cells for 24 h at 25°C before imaging. Eight independent experiments were performed. The data were plotted with Prism (GraphPad Software, Inc.).

### Cell viability assay.

Cell viability was assessed as described by Zhang et al. (64) with the following modifications. Freshly dissolved hit compounds in DMSO were added to IMR-90, cells and cell viability was assessed after for 24 h at 25°C by a PrestoBlue cell viability reagent (Life Technologies). Ten independent experiments were performed using serum-free Eagle’s minimum essential medium (Wisent) and the same medium supplemented with 10% fetal bovine serum (FBS) (complete medium). The data were plotted using nonlinear least-squares fitting to a dose-response model in Prism (GraphPad software).

### Evaluating *in vitro* esterase activity using ACC.

ACC was used as a substrate to compare the specific activities of PelAΔ46_Nhis6_ and PelAΔ46_Chis6_ as follows. The assay was performed using a 96-well black-bottom plate (Thermo Fisher). Enzyme reaction wells had 5 μL of enzyme and 90 μL of reaction buffer. Enzyme blank reaction wells had 95 μL of reaction buffer. The assay was initiated by the addition of 5 μL ACC solubilized in DMSO to all wells. Using a SpectraMax M2 (Molecular Devices) plate reader, the RFU was measured at 20-s intervals over 5 min at room temperature using an λ_em_ of 386 nm and λ_ex_ of 447 nm. Reaction mixtures were maintained at a final volume of 100 μL with 3.1 μM PelAΔ46_Nhis6_ or 0.4 μM PelAΔ46_Chis6_, 2.5 μM ACC, and 5% (vol/vol) DMSO. The background hydrolysis rate was monitored and subtracted from the enzyme-catalyzed reactions. A standard curve was generated using the product of the reaction, 7-hydroxycoumarin-3-carboxylic acid, to calculate the specific activity. Three independent experiments (*n* = 3 wells per experiment) were performed.

To generate a kinetic curve, various concentrations of ACC and 2 μM PelAΔ46_Chis6_ were used as described above and the standard curve was used to calculate the rate. Kinetic parameters were calculated using the general Michaelis-Menten model in Prism (GraphPad Software, Inc.).

### Evaluating *in vitro* esterase activity using AMMU.

AMMU was used as a substrate to generate IC_50_ curves as follows. Frozen PelAΔ46_Chis6_ aliquots were thawed on ice for 5 min and diluted in reaction buffer to create a PelA master mix. Hit compounds and AMMU were freshly dissolved in DMSO prior to use. A 25% (vol/vol) DMSO solution was freshly made using reaction buffer. A mixing plate was generated using a 96-well polypropylene plate, wherein each row contained 20 μL of reaction buffer, 20 μL of PelA master mix, serially diluted hit compound in 25% (vol/vol) DMSO, and 10 μL of 4 mM AMMU. To begin the assay, 2.5 μL of serially diluted hit compound was added to the reaction buffer and PelA master mix for the enzyme blank and enzyme reactions, respectively. To initiate the reaction, 2.5 μL of AMMU was added to the enzyme blank and enzyme reaction solutions, for a total reaction volume of 25 μL. From the mixing plate, 10 μL of solution was added to a 384-well, black, flat-bottom assay plate (Corning; 3820) in duplicate and centrifuged at 3,000 rpm for 30 s. Using a Synergy Neo2 (BioTek) plate reader, the RFU was measured at 30-s intervals over 10 min at room temperature using an λ_em_ of 330 nm and λ_ex_ of 450 nm. Final reaction mixtures were maintained at a final volume of 10 μL with 1 μM PelAΔ46_Chis6_, 400 μM AMMU, and 12.5% (vol/vol) DMSO. The background hydrolysis rate was monitored and subtracted from the enzyme-catalyzed reactions. The percentage of PelA esterase activity was calculated using the RFU per minute of enzyme reactions with no hit compound present. IC_50_ values were calculated using nonlinear least-squares fitting to a dose-response model in Prism (GraphPad software).

For kinetic characterization, the assay was performed as described above with the following modifications. The mixing plate contained a row with 10 μL of hit compound at 25% (vol/vol) DMSO and 10 μL of AMMU serially diluted in 50% (vol/vol) DMSO. In the mixing plate, 2.5 μL of hit compound was added to wells with reaction buffer and PelA master mix for enzyme blank and enzyme reactions, respectively, sealed with adhesive foil (VWR), and left to incubate for 2 h at room temperature. The reaction was initiated as indicated above. Final reaction mixtures had 1 μM PelAΔ46_Chis6_ and 7.5% (vol/vol) DMSO. Prism (GraphPad Software Inc.) was used for all kinetic analyses and the sum of squares *F* test. Apparent *V*_max_ and *K_m_* parameters were determined by nonlinear regression fitting the initial velocities (*v_i_*) to the Michaelis-Menten model:

$$v_{\text{i}}=\frac{V_{\text{max}}\left[S\right]}{K_{\text{m}}+\left[S\right]}$$

Lineweaver-Burk analysis was conducted by fitting the double reciprocal form of the Michaelis-Menten equation to initial velocity data using linear regression:

$$\frac{1}{v_{i}}=\left(\frac{K_{m}}{V_{\text{max}}}\right)\frac{1}{\left[S\right]}+\frac{1}{V_{\text{max}}}$$

Finally, the inhibitor constant, *K_i_*, was calculated by globally fitting the initial velocity data to a noncompetitive inhibition model:

$$v_{i}=\frac{V_{\text{max}}\left[S\right]}{K_{m}+\left[S\right]\left(1+\frac{\left[I\right]}{K_{i}}\right)}$$

### Reversibility test using gel filtration.

To assess the reversibility of the compounds, 0.5-mL Zeba spin desalting columns with a 7,000-molecular-weight cutoff (MWCO) (Thermo Scientific) were used as follows. The Zeba spin desalting columns were prepared and buffer exchanged into reaction buffer as per the manufacturer’s instructions. Frozen PelAΔ46_Chis6_ aliquots, hit compounds, AMMU, and 25% (vol/vol) DMSO were prepared as outlined above for the AMMU assay. A 96-well polypropylene conical-bottom plate (Thermo Scientific) serving as a mixing plate was used to set up three reaction types: enzyme-only, enzyme plus compound control, and reversibility test. To one row of wells in the mixing plate, 40 μL of PelA master mix was added. To three wells for each of the enzyme-only reaction and reversibility test, 5 μL of 25% (vol/vol) DMSO and 5 μL of 0.25 mM hit compound in 25% (vol/vol) DMSO were added, respectively. From wells in the mixing plate, 45 μL corresponding to the enzyme-only reaction and reversibility test and 40 μL of PelA master mix corresponding to the reversibility test were added to the resin beds of a total of nine Zeba spin desalting columns per compound. As per the manufacturer’s instructions, the Zeba spin desalting columns were centrifuged at 1,500 × *g* for 2 min to perform the gel filtration and collect the eluates. The eluates were transferred back to the mixing plate, across a second row of wells. To the enzyme plus compound control reaction mixtures, 5 μL of 0.25 mM hit compound in 25% (vol/vol) DMSO was added.

The *in vitro* esterase activities of the three reaction mixtures were evaluated using the AMMU assay described above. To a third row of wells in the mixing plate, 10 μL of 4 mM AMMU was added. To initiate the reaction, 5 μL of 4 mM AMMU was added to the eluates in the second row of the mixing plate, from which two aliquots of 10 μL were transferred to a 384-well, black, flat-bottom assay plate (Corning; 3820). The rest of the AMMU assay was performed as outlined above. Final reaction mixtures were maintained at a final volume of 10 μL with 1 μM PelAΔ46_Chis6_, 400 μM AMMU, and 12.5% (vol/vol) DMSO. Three independent experiments (*n* = 2 wells per experiment) were performed.
