# Supplementary material for: Small Molecule Inhibition of an Exopolysaccharide Modification Enzyme is a Viable Strategy To Block Pseudomonas aeruginosa Pel Biofilm Formation
Source: Microbiol Spectr. 2023 Apr 26;11(3):e00296-23. doi: 10.1128/spectrum.00296-23 (PMC10269871; doi:10.1128/spectrum.00296-23)

Supplementary Table 1: Bacterial strains used in this study.

Strains	Genotype or relevant characteristics[1]	Source
Pseudomonas aeruginosa		
PAO1	Wild type strain from Colin Manoil, genome resequenced	(1)
PAO1 Δ*wspF* Δ*psl* P_{BAD}*pel*	In-frame deletion of *wspF*, polar deletion of *pslBCD*, *araC*-P_{BAD} inserted upstream of *pelABCDEFG*	(2)
JJH885	PAO1 Δ*wspF* Δ*psl* P_{BAD}*pel* Δ*pelF*	(3)
JJH879	PAO1 Δ*wspF* Δ*psl* P_{BAD}*pel* Δ*pelA*	(4)
JDR82	Gen^r, JJH879 *attTn7*::miniTn7T-Gm	(4)
LSM30	Gen^r, JJH879 *attTn7*::miniTn7T2.1-Gm-GW::*araC*-P_{BAD}::*pelA*	(5)
JDR47	Gen^r, JJH879 *attTn7*::miniTn7T2.1-Gm-GW::*araC*-P_{BAD}::*pelA*^{D528A}	This study
PAO1 Δ*pelF* P_{BAD}*psl*	In-frame deletion of *pelF*, *araC-*P_{BAD} inserted upstream of *pslA*	(6)
PAO1 Δ*wspF* Δ*pelF* P_{BAD}*psl*	In-frame deletion of *wspF* and *pelF*, *araC-*P_{BAD} inserted upstream of *pslA*	This study
PA14	Wild type strain	(2)
PA14 Δ*wspF*	*wspF,* nonpolar mutation	(7)
Bacillus cereus		
ATCC 10987	Wild type strain	A.J. Clarke
Escherichia coli		
TOP10	Str^R, F^- mcrAΔ(mrr-*hsd*RMS-*mcr*BC) Φ80*lacZ* ΔM15 Δ*lac*X74 *rec*A1 *ara*D139 Δ(ara leu) 7697 *gal*U *gal*K *rps*L *end*A1 *nup*G	Invitrogen
BL21CodonPlus (DE3)-RP	Kan^R, F^- *ompT hsdS*(rB^- mB^-)*dcm*$^+$ Tet^r galλ (DE3) *end*AHte *met*A::Tn5(Kan^r) [*arg*U *ile*Y *leu*W Cam^r]	Stratagene
SM10 (λ_{pir})	Kan^R, Tet^R. *thi thr leu tonA lacY supE recA::RP4-2-Tc::Mu* K_m λ_{pir}	(8)
XL10-Gold	Tet^R, Δ(*mcrA*)183 Δ(*mcrCB-hsdSMR-mrr*)173 *endA1 supE*44 *thi*-1 *recA1 gyrA96 rel*A1 *lac* Hte [F' *proAB lacI*^{q} ZΔ*M15* Tn*10* (Tet^R) Amy Cam^R]	Agilent Technologies

[1]Abbreviations for antibiotic selection: Gen, gentamicin; Str, streptomycin; Tet, tetracycline; Cam, chloramphenicol; Kan, kanamycin.

Supplementary Table 2: Plasmids used in this study.

Plasmids	Genotype or relevant characteristics[1]	Source
**Protein production**		
pET-24a(+)	Kan^R, IPTG-inducible expression vector	Novagen
pER-PelA$^{47\text{-}948}$	PelA encoding the full-length protein excluding the predicted signal sequence. Expressed in pET-24a	This study
pNA*pelA*$^{\Delta 46}$	pET28a based *pelA* expression vector	(9)
**Complementation analysis**		
pUC18-miniTn7T2.1-Gm-GW	Amp^R, Gen^R, Cam^R, miniTn7 with transcriptional terminators flanking the Tn7 transposon; Gateway destination vector	(10)
pTNS2	Amp^R, helper plasmid encoding *tnsABCD*	(11)
pCAS4	Gen^R, Amp^R, pUC18-miniTn7T2.1-Gm-GW with *araC*-P_{BAD}::*pelA* in the Gateway cloning site	(5)
pJDR45	Gen^R, Amp^R, pCAS4 with P_{BAD}::*pelA*^{D528A}	This study
**Allelic exchange vectors**		
pEX18Gm	Gen^R, suicide vector for allelic exchange in *P. aeruginosa*, SacB, lacZα	(8)
pEX18Gm::Δ*wspF*	Gen^R, in-frame *wspF* deletion construct	(12)

[1]Abbreviations for antibiotic selection: Amp, ampicillin; Cam, chloramphenicol; Kan, kanamycin; Gen, gentamicin.

Supplementary Table 3: Primers used in this study.

Primer name or identifier	Sequence[1]
**Vectors for recombinant protein production**	
oER5_PelA47ChisF-NdeI	GGG **CAT ATG** <u>GGC GGG CCG TCC AGC GTG GCG</u>
oER6_PelA948ChisR-XhoI	GGG **CTC GAG** <u>GCG GCA GAC GAG TTG GCC ATC GCG</u>
**Sequencing primers**	
GW7_wspFupF	CGT **GAA TTC** <u>TGA ACG TCC TGC TGG TGC CGGA</u>
GW10_wspFdownR	TCT **AAG CTT** <u>CTT CCT TGG TCG ACA GGA CGA T</u>
oER7_PelA47_int1	<u>GCG GTG GCC GTG GAG TCG ATC</u>
oER8_PelA47_int2	<u>CTC GAC GAA AAG GTG CCG GTG</u>
oER9_PelA47_int3	<u>CAG GTG CTG GAG GAC TTC ATC</u>
oER10_PelA47_int4	<u>CAG TAC TAC GCG CCG ATC ATC</u>
oJDR33_*araC-ParaBAD*-500F01-SEQ	<u>GGT GCG CTT CAT CCG GGC G</u>
oJDR34_*araC-ParaBAD*-1000F01-SEQ	<u>GCA TTC TGT AAC AAA GCG G</u>
oJDR35_PelA-308R01-SEQ	<u>CGA TGG CGG CGG CGT CG</u>
oJDR36_PelA-770R01-SEQ	<u>GCG GCA GGT AGT CGA TGG CG</u>
oJDR37_PelA-1308R01-SEQ	<u>GCC GGC AGG TCG CGG ATC CG</u>
oJDR38_PelA-1808R01-SEQ	<u>CTG CCA GAA GAA CGG ATG G</u>
oJDR39_PelA-2308R01-SEQ	<u>GGT GCA TGG TGC GGA TCG</u>
oJDR40_PelA-2808R01-SEQ	<u>CAT CGC GCA CCT GCT CCA TC</u>
oJJH371_PTn7R	<u>CAC AGC ATA ACT GGA CTG ATT TC</u>
oJJH372_PTn7L	<u>ATT AGC TTA CGA CGC TAC ACC C</u>
oJJH374_PglmS-up	<u>CTG TGC GAC TGC TGG AGC TGA</u>
oJJH373_PglmS-down	<u>GCA CAT CGG CGA CGT GCT CTC</u>
**Site-directed mutagenesis**	
oJDR41_PelA-D528A-F01	<u>GCC ACC GTG CAC ATC</u> gcc <u>GGC GAC GGC TTC GTC</u>
oJDR42_PelA-D528A-R01	<u>GAC GAA GCC GTC GCC</u> ggc <u>GAT GTG CAC GGT GGC</u>

[1]Restriction and attachment sites are bolded; regions complementary to the target amplicon are underlined; regions of reverse complementarity (to facilitate splicing) are italicized; lowercase letters denote a nucleotide substitution.

Supplementary Information References:

1. **Harrison JJ**, **Almblad H**, **Irie Y**, **Wolter DJ**, **Eggleston HC**, **Randall TE**, **Kitzman JO**, **Stackhouse B**, **Emerson JC**, **Mcnamara S**, **Larsen TJ**, **Shendure J**, **Hoffman LR**, **Wozniak DJ**, **Parsek MR**. 2020. Elevated exopolysaccharide levels in *Pseudomonas aeruginosa* flagellar mutants have implications for biofilm growth and chronic infections. PLoS Genet **16**:1–22.
2. **Colvin KM**, **Alnabelseya N**, **Baker P**, **Whitney JC**, **Howell PL**, **Parsek MR**. 2013. PelA Deacetylase Activity Is Required for Pel Polysaccharide Synthesis in Pseudomonas aeruginosa. J Bacteriol **195**:2329–2339.
3. **Whitfield GB**, **Marmont LS**, **Ostaszewski A**, **Rich JD**, **Whitney JC**, **Parsek MR**, **Harrison JJ**, **Howell PL**. 2020. Pel Polysaccharide Biosynthesis Requires an Inner Membrane Complex Comprised of PelD, PelE, PelF, and PelG. J Bacteriol **202**:471–18.
4. **Razvi E**, **Whitfield GB**, **Reichhardt C**, **Dreifus JE**, **Willis AR**, **Gluscencova OB**, **Gloag ES**, **Awad TS**, **Rich JD**, **da Silva DP**, **Bond W**, **Le Mauff F**, **Sheppard DC**, **Hatton BD**, **Stoodley P**, **Reinke AW**, **Boulianne GL**, **Wozniak DJ**, **Harrison JJ**, **Parsek MR**, **Howell PL**. 2023. Glycoside hydrolase processing of the Pel polysaccharide alters biofilm biomechanics and *Pseudomonas aeruginosa* virulence. npj Biofilms Microbiomes **X**:X–X.
5. **Marmont LS**, **Whitfield GB**, **Rich JD**, **Yip P**, **Giesbrecht LB**, **Stremick CA**, **Whitney JC**, **Parsek MR**, **Harrison JJ**, **Howell PL**. 2017. PelA and PelB form a modification and secretion complex essential for Pel polysaccharide-dependent biofilm formation in *Pseudomonas aeruginosa*. J Biol Chem **292**:19411–19422.
6. **Baker P**, **Whitfield GB**, **Hill PJ**, **Little DJ**, **Pestrak MJ**, **Robinson H**, **Wozniak DJ**, **Howell PL**. 2015. Characterization of the *Pseudomonas aeruginosa* Glycoside Hydrolase PslG Reveals That Its Levels Are Critical for Psl Polysaccharide Biosynthesis and Biofilm Formation*. J Biol Chem **290**:28374–28387.
7. **Jennings LK**, **Storek KM**, **Ledvina HE**, **Coulon C**, **Marmont LS**, **Sadovskaya I**, **Secor PR**, **Tseng BS**, **Scian M**, **Filloux A**, **Wozniak DJ**, **Howell PL**, **Parsek MR**. 2015. Pel is a cationic exopolysaccharide that cross-links extracellular DNA in the *Pseudomonas aeruginosa* biofilm matrix. Proc Natl Acad Sci USA **112**:11353–11358.
8. **Hoang TT**, **Karkhoff-Schweizer RR**, **Kutchma AJ**, **Schweizer HP**. 1998. A broad-host-range Flp-FRT recombination system for site-specific excision of chromosomally-located DNA sequences: application for isolation of unmarked *Pseudomonas aeruginosa* mutants. Gene **212**:77–86.
9. **Colvin KM**, **Alnabelseya N**, **Baker P**, **Whitney JC**, **Howell PL**, **Parsek MR**. 2013. PelA Deacetylase Activity Is Required for Pel Polysaccharide Synthesis in *Pseudomonas aeruginosa*. J Bacteriol **195**:2329–2339.
10. **Zhao K**, **Tseng BS**, **Beckerman B**, **Jin F**, **Gibiansky ML**, **Harrison JJ**, **Luijten E**, **Parsek MR**, **Wong GCL**. 2013. Psl trails guide exploration and microcolony formation in Pseudomonas aeruginosa biofilms. Nature **497**:388–392.
11. **Choi K-H**, **Schweizer HP**. 2006. mini-Tn7 insertion in bacteria with single attTn7 sites: example Pseudomonas aeruginosa. Nat Protoc **1**:153–161.
12. **Limoli DH**, **Whitfield GB**, **Kitao T**, **Ivey ML**, **Davis MR Jr**, **Grahl N**, **Hogan DA**, **Rahme LG**, **Howell PL**, **O'Toole GA**, **Goldberg JB**. 2017. *Pseudomonas aeruginosa* Alginate Overproduction Promotes Coexistence with *Staphylococcus aureus* in a Model of Cystic Fibrosis Respiratory Infection. mBio **8**:1–18.

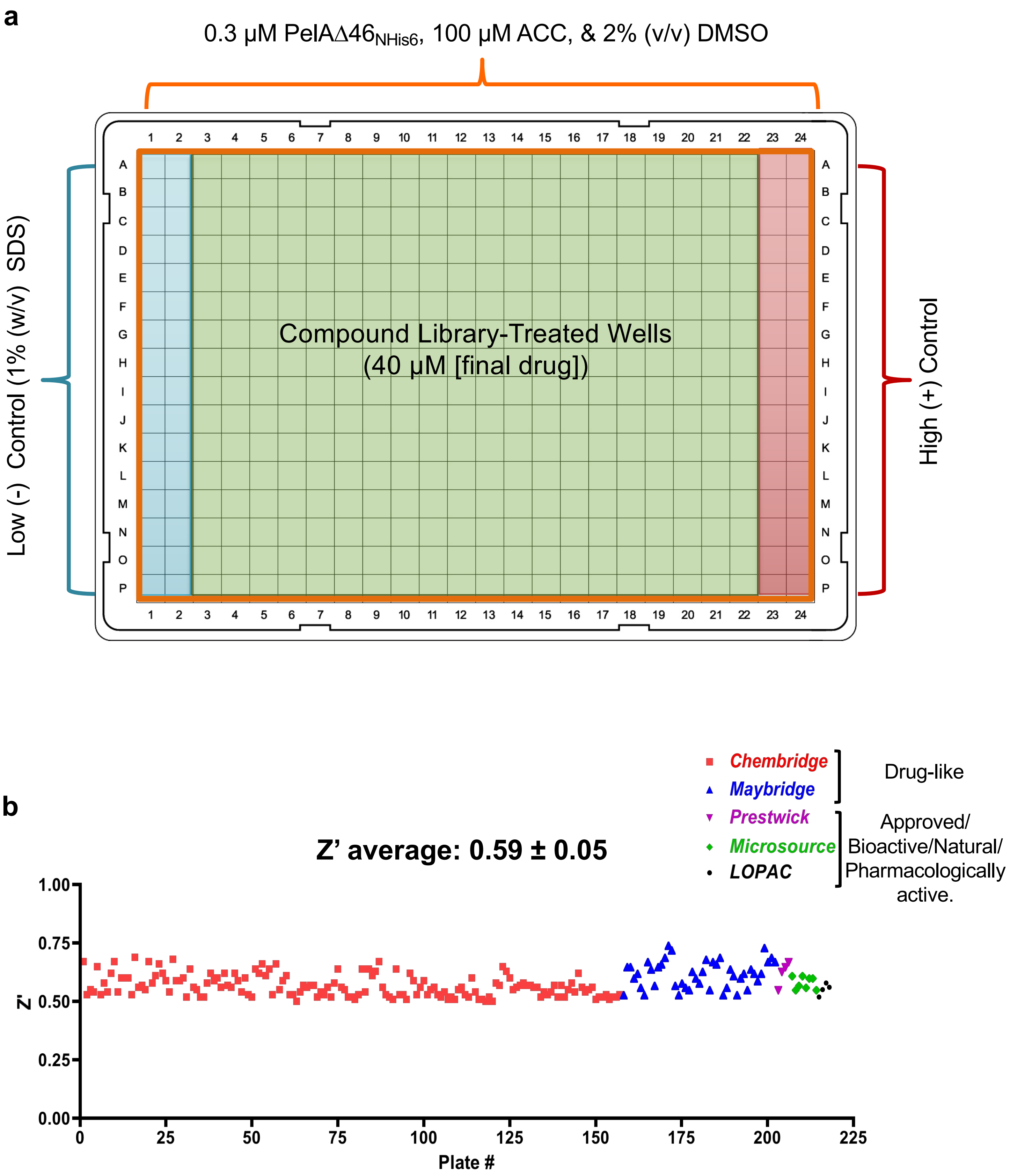

Supplementary Figure 1: High-throughput screen assay setup and quality. (A) Schematic of 384-well assay plate set up. (B) Calculated Z' score categorized by chemical library across 217 plates used for screen. ACC, 7-acetoxycoumarin-3-carboxylic acid; DMSO, dimethyl sulfoxide; SDS, sodium dodecyl sulfate.

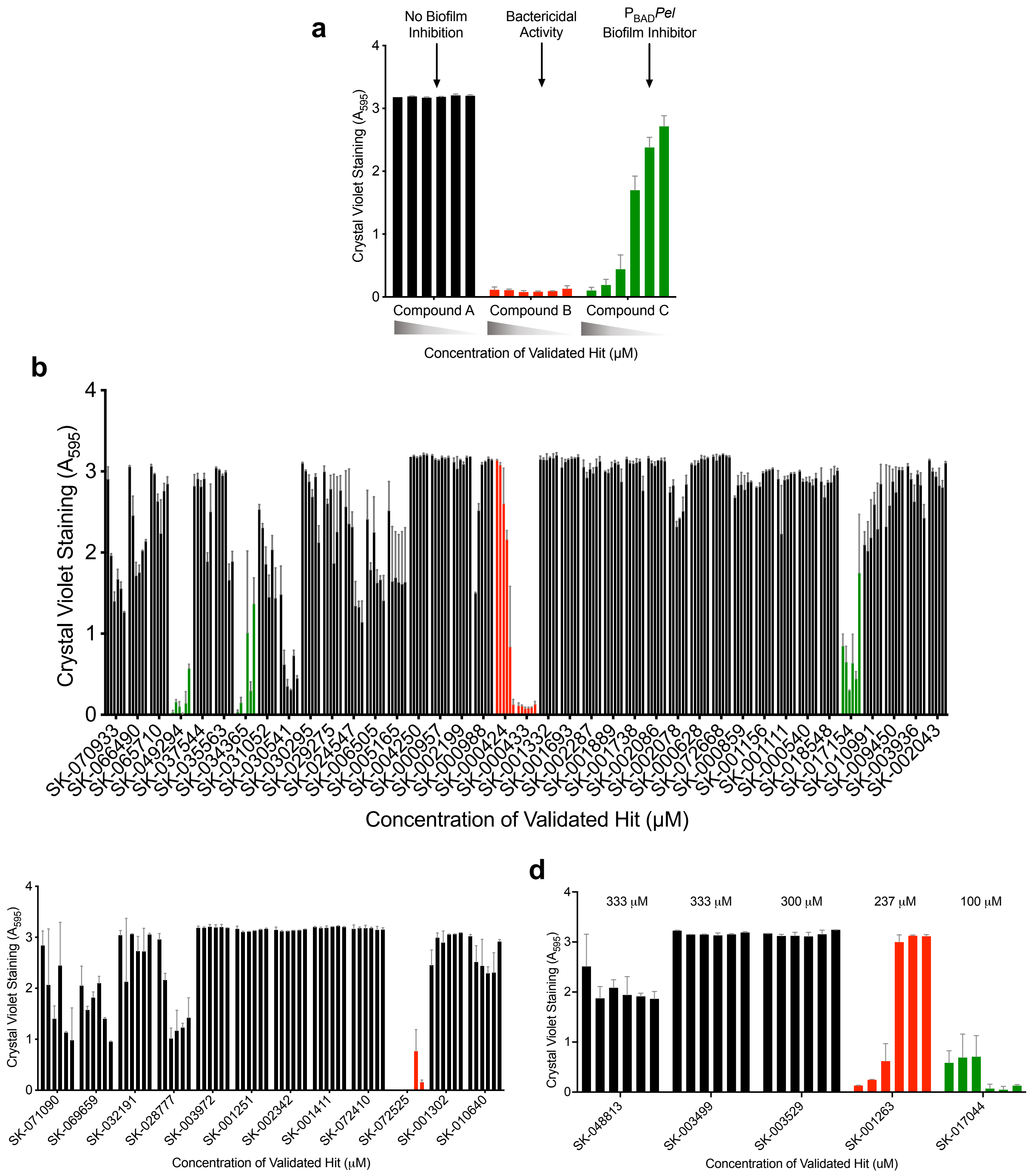

Supplementary Figure 2: Identification of four validated hit compounds able to inhibit P_{BAD} *pel* biofilms (A) Classification of validated hits subjected to secondary crystal violet assay. Black bars indicate no biofilm inhibition, red bars bactericidal activity, and green bars P_{BAD}*pel* biofilm inhibitors. Secondary screening results of validated hits starting at a final concentration of (B) 1000 μM (C) 500 μM. (D) 333 μM, 300 μM, 237 μM, and 100 μM indicated above respective data. All validated hits were added to generate 6 point 2-fold serial dilutions starting at the indicated initial final concentration. Error bars represent standard error of the mean of two independent trials. P_{BAD}*pel*, PAO1 Δ*wspF* Δ*psl* P_{BAD}*pel*.

a

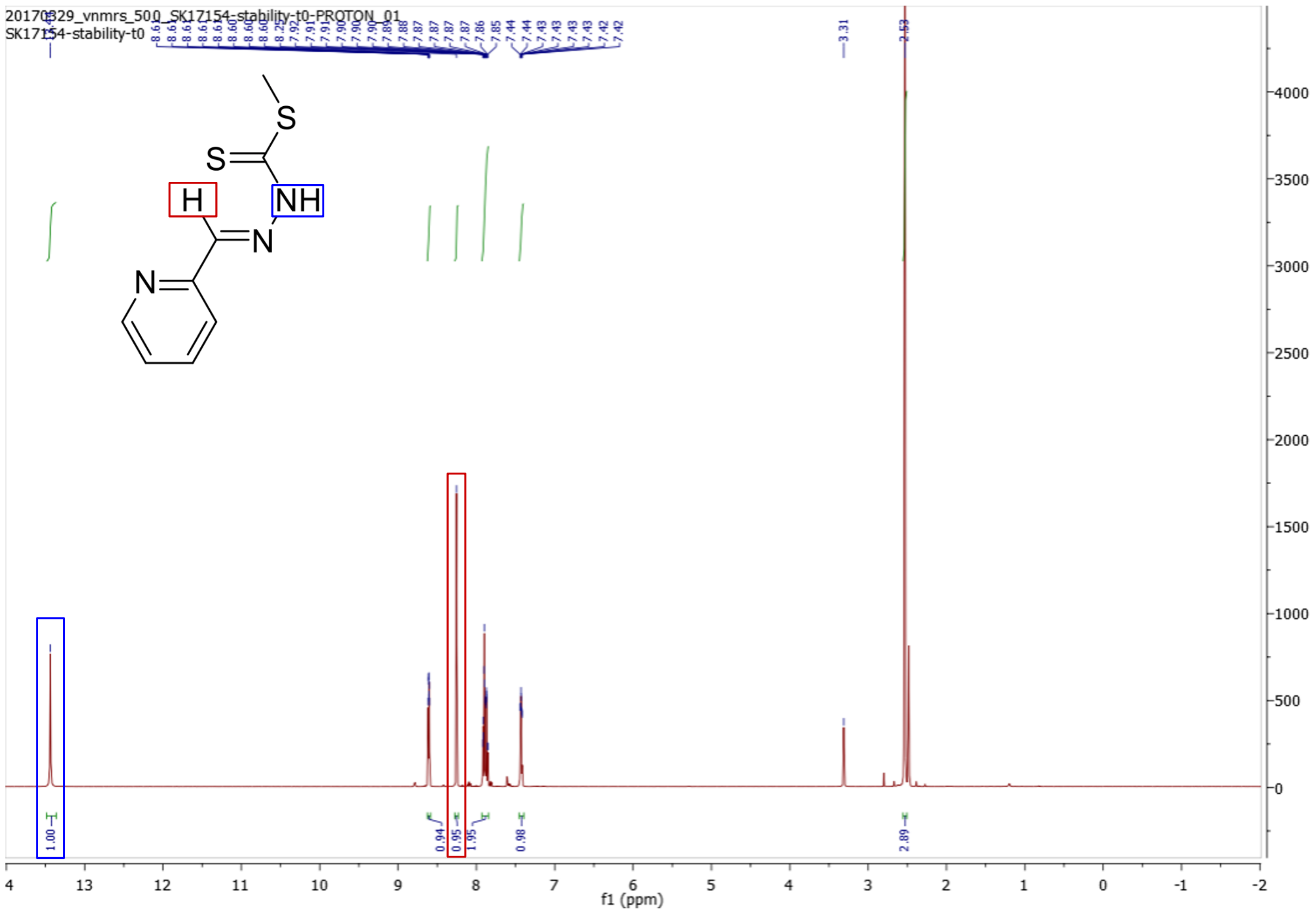

b

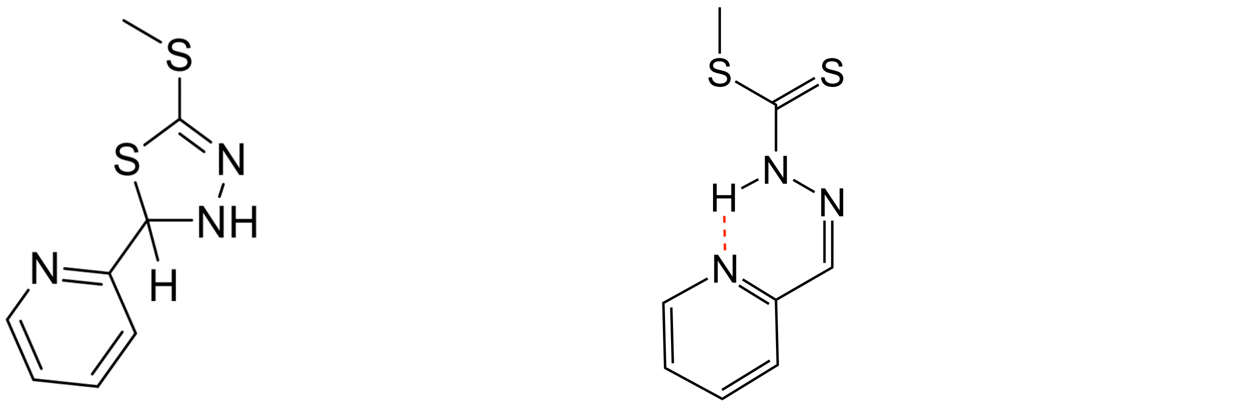

SK-017154 **SK-017154-O**

Supplementary Figure 3: Stable precursor of SK-017154, SK-017154-O, inhibits PA14 biofilm formation. (A) ^{1}H NMR spectra of material purchased from the supplier which was labelled as SK-017154. (B) Structure provided by the supplier for SK-01754 and the structure of 2-pyridinecarboxaldehyde Schiff base of S-methyldithiocarbazate (SK-01754-O) illustrating the hydrogen bond between the pyridyl nitrogen and the N-H.

c

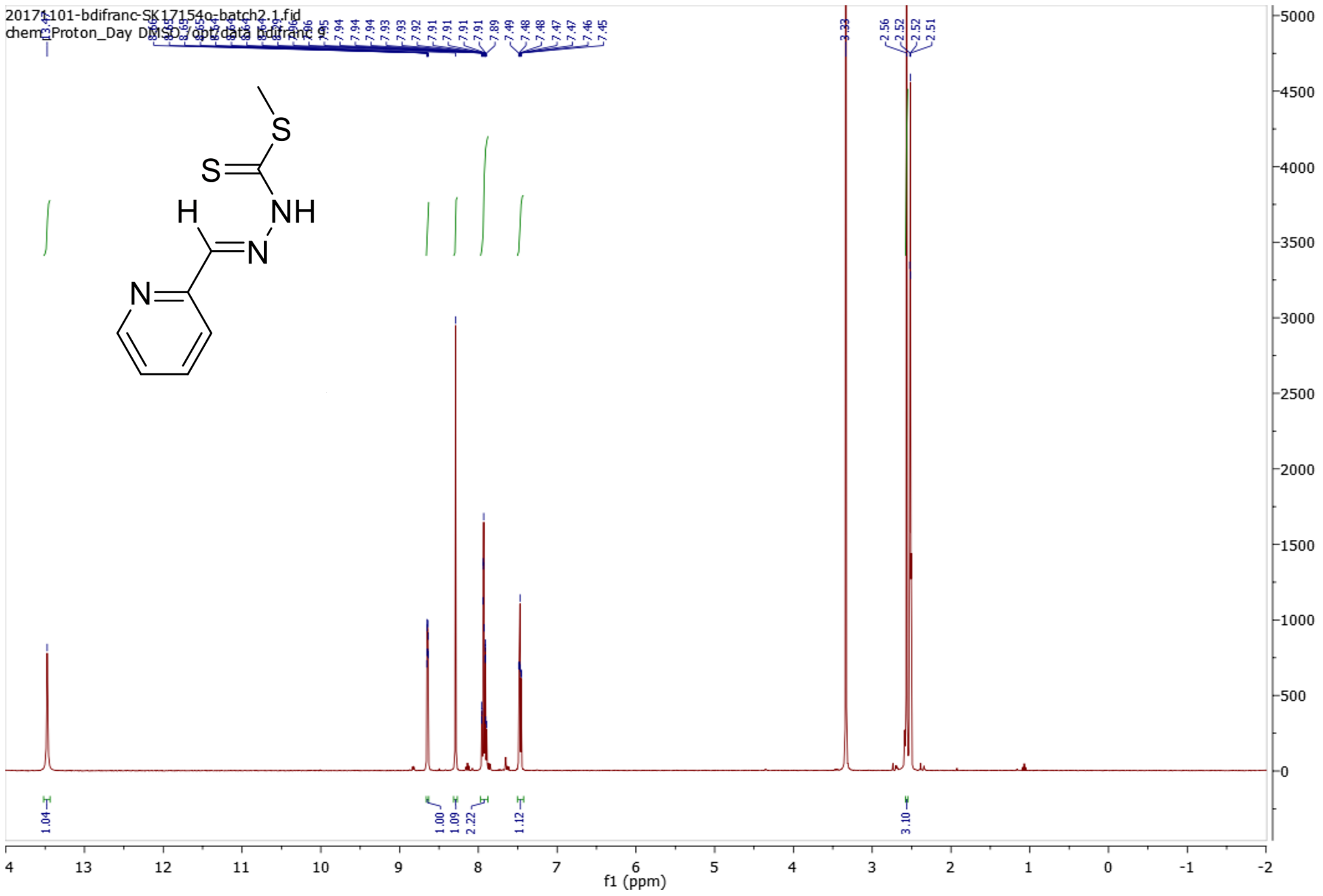

d

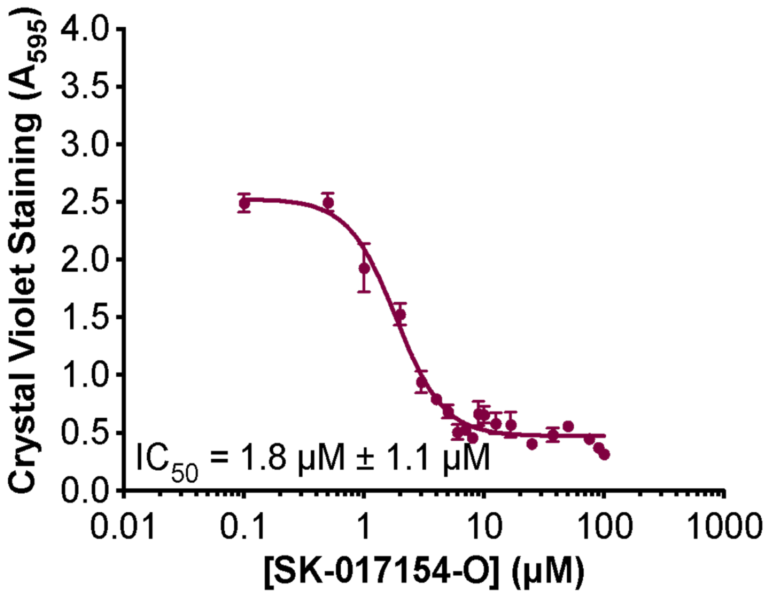

Supplementary Figure 3 (continued): Stable precursor of SK-017154, SK-017154-O, inhibits PA14 biofilm formation. (C) ^{1}H NMR spectra of synthesized SK-017154-O. (D) Secondary *ex vivo* crystal violet assay results to PA14 using SK-017154-O. Error bars represent standard error of the mean of six independent trials.

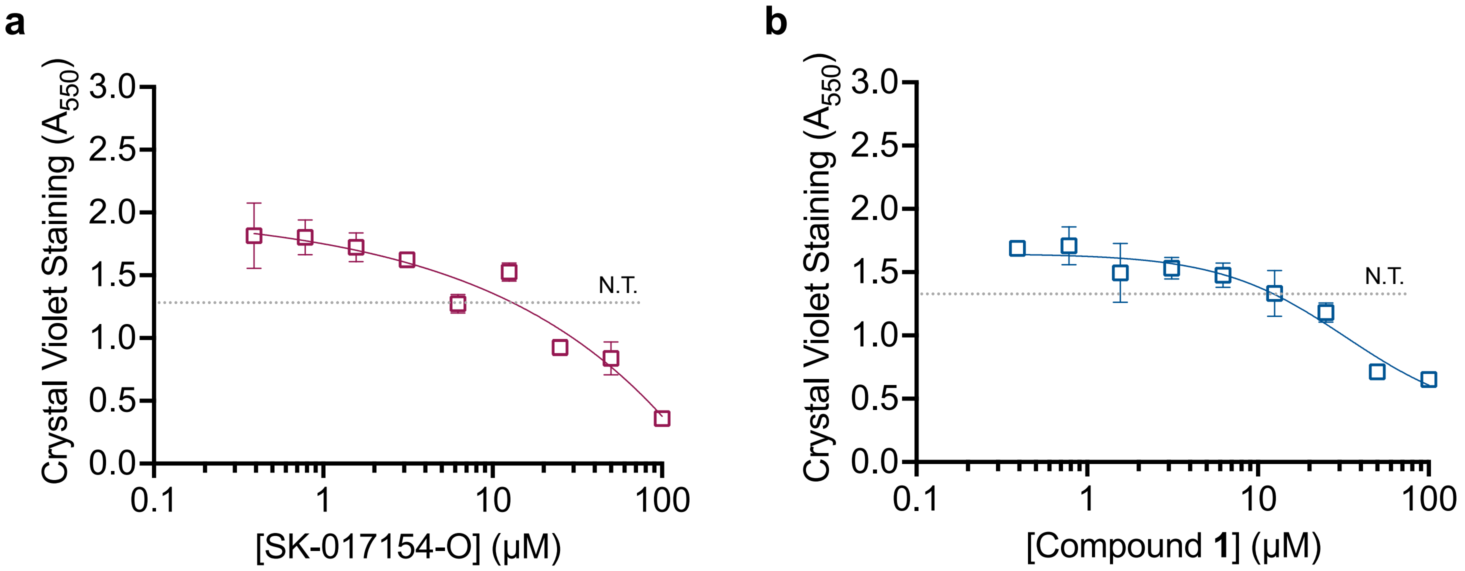

Supplementary Figure 4: SK-017154-O and Compound 1 can inhibit *B. cereus* Pel dependent biofilms. Secondary *in vitro* biofilm inhibition assay using (A) SK-017154-O and (B) Compound **1**. Error bars represent standard error of the mean of three independent trials. N.T., average A_{550} of untreated control wells.

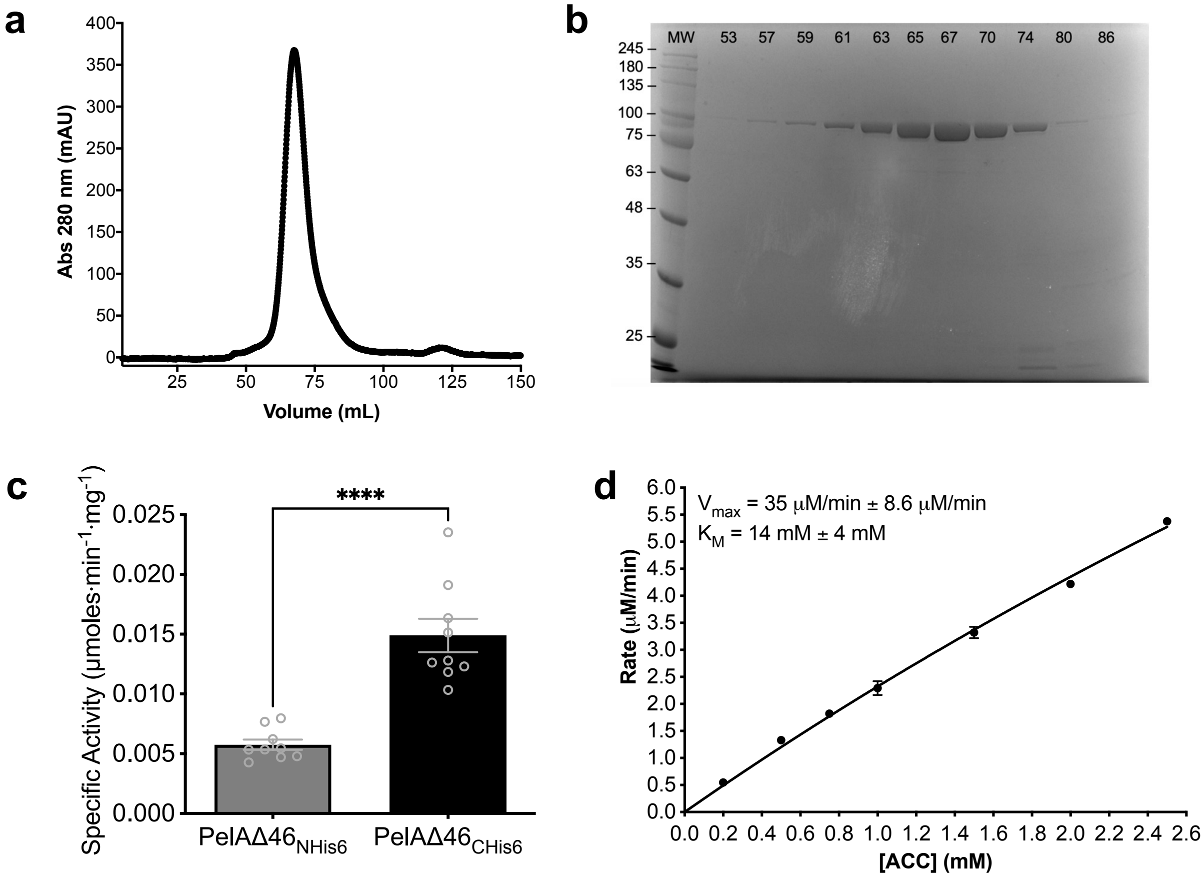

Supplementary Figure 5: PelAΔ46$_{Chis6}$ is less degradative and more active in ACC assay than PelAΔ46$_{Nhis6}$ (A) Gel filtration trace of PelAΔ46$_{Chis6}$. (B) Coomassie-stained SDS-PAGE corresponding to the individual peak. Elution fractions are indicated at the top. The molecular weight of PelAΔ46$_{Chis6}$ is101.1 kDa. (C) Specific activity of PelAΔ46$_{Nhis6}$ or PelAΔ46$_{Chis6}$ hydrolysis of ACC. Error bars represent standard error of the mean of nine independent trials. Statistical significance was evaluated using an unpaired T-test with equal standard deviation. ****, $P<0.0001$. (D) PelAΔ46$_{Chis6}$ enzymology using ACC as substrate. Steady-state kinetics using 0.2 mM to 2.5 mM ACC and 2 μM PelAΔ46$_{CHis6}$. Error bars represent standard error of the mean of three independent trials. MW, molecular weight marker in kDa; ACC, 7-acetoxycoumarin-3-carboxylic acid.

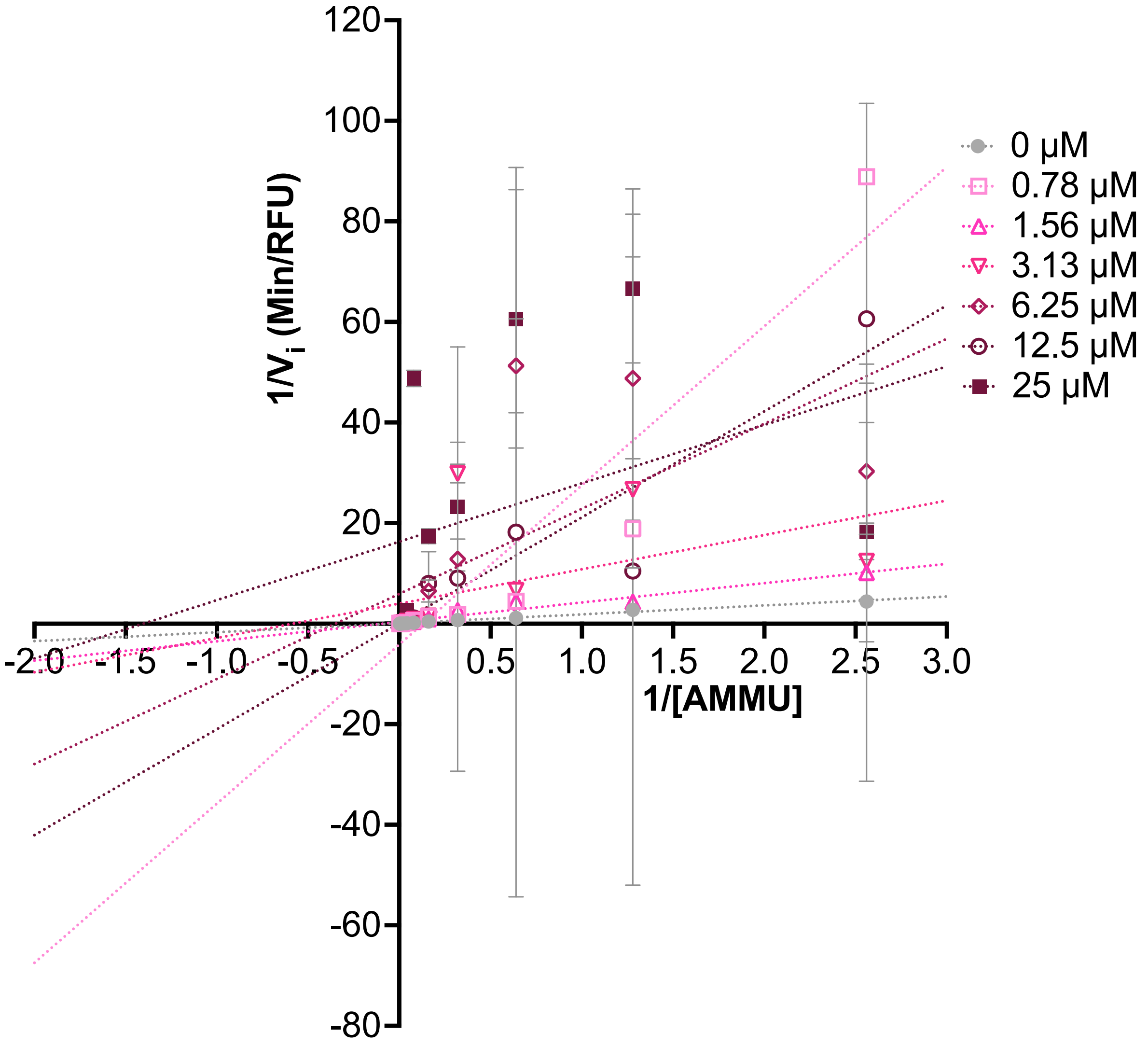

Supplementary Figure 6: Lineweaver-Burk plot of SK-017154-O inhibition kinetics in Fig. 8B
Error bars represent the standard deviation of two independent trials. RFU, residual fluorescence units; AMMU, acetoxymethyl-4-umbelliferone.

Supplementary Figure 7: NMR analyses of synthesized compounds. 2-pyridinecarboxaldehyde *S*-methyldithiocarbazate (SK17154-O) (A) ^{1}H NMR. (B) ^{13}C NMR. Benzaldehyde *S*-methyldithiocarbazate (1) (C) ^{1}H NMR. (D) ^{13}C NMR. Benzaldehyde *S*-ethyldithiocarbazate (9) (E) ^{1}H NMR. (F) ^{13}C NMR. Benzaldehyde *S*-benzyldithiocarbazate (10) (G) ^{1}H NMR. (H) ^{13}C. Benzaldehyde methylcarbazate (11) (I) ^{1}H NMR. (J) ^{13}C. Methyl 2-phenylethyl carbamodithioate (12) (K) ^{1}H NMR. (L)

a 2-pyridinecarboxaldehyde *S*-methyldithiocarbazate (SK17154-O) ^{1}H NMR

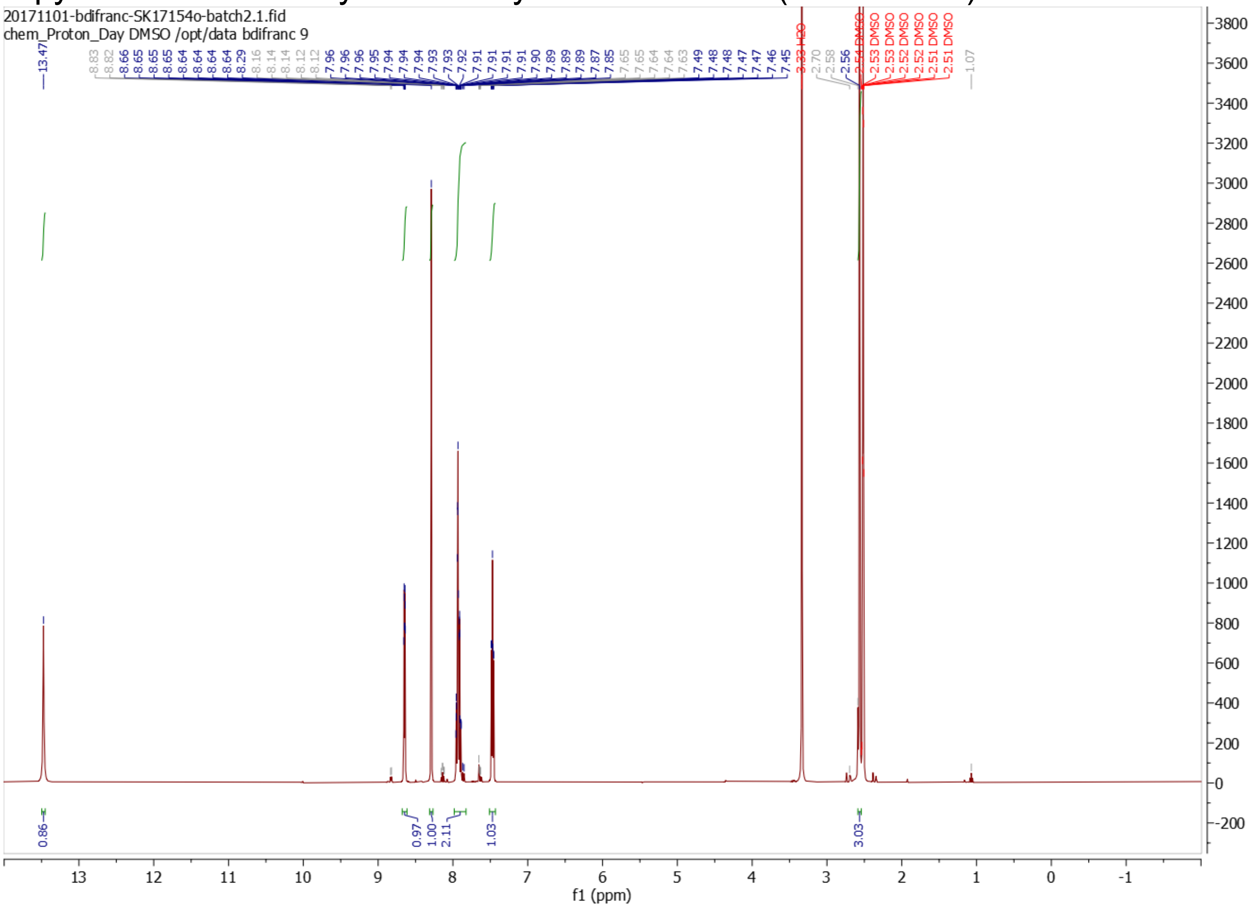

b 2-pyridinecarboxaldehyde *S*-methyldithiocarbazate (SK17154-O) ^{13}C NMR

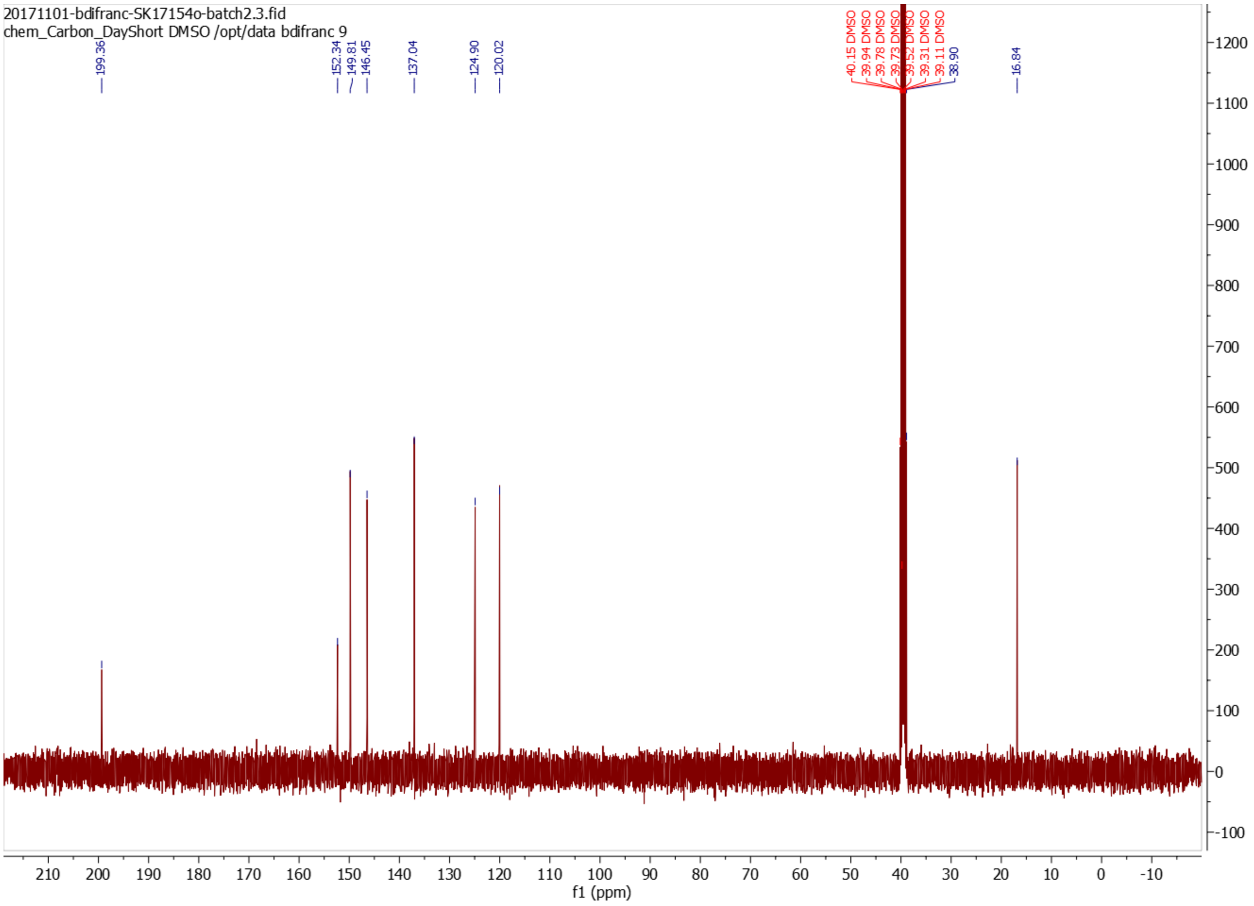

c Benzaldehyde *S*-methyldithiocarbazate (1) ^{1}H NMR

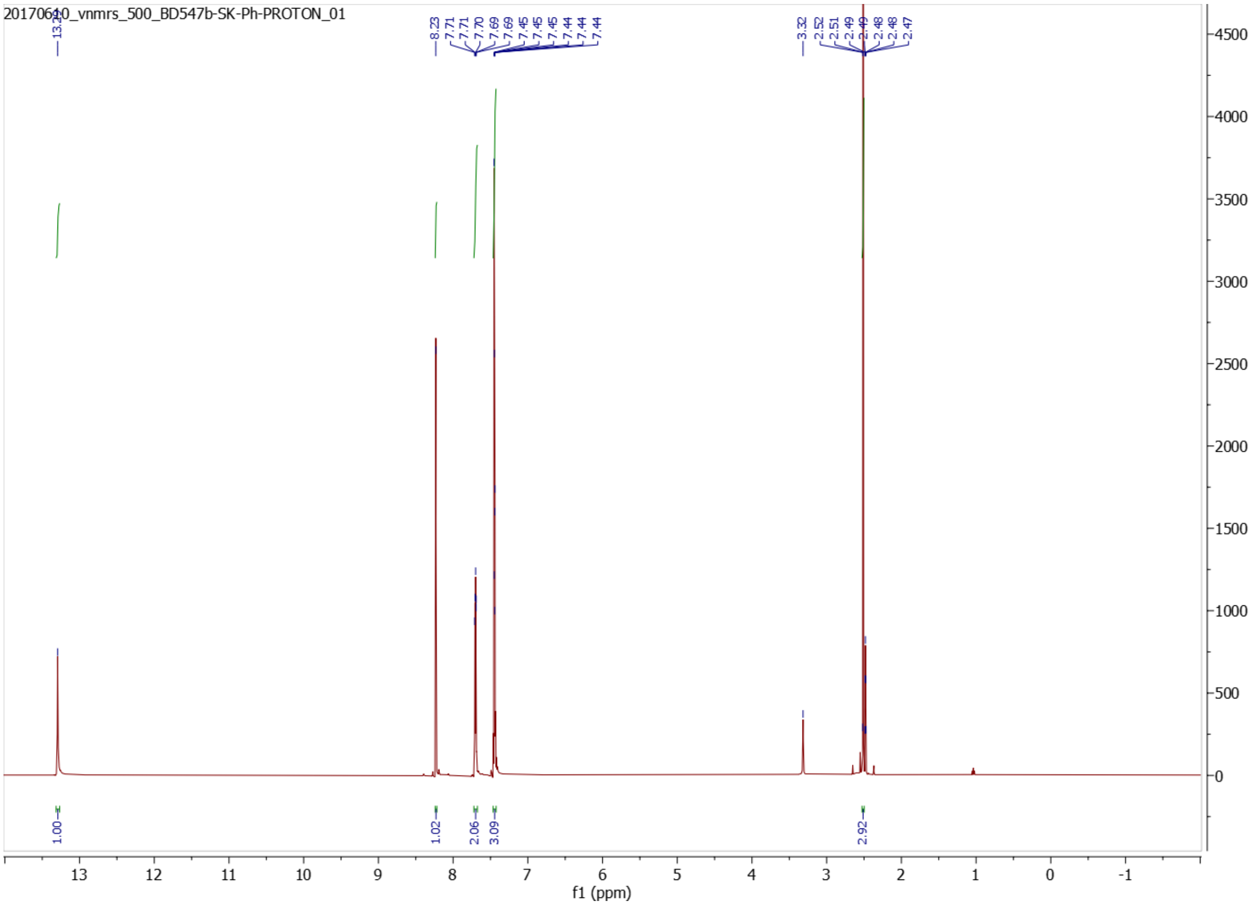

d Benzaldehyde *S*-methyldithiocarbazate (1) ^{13}C NMR

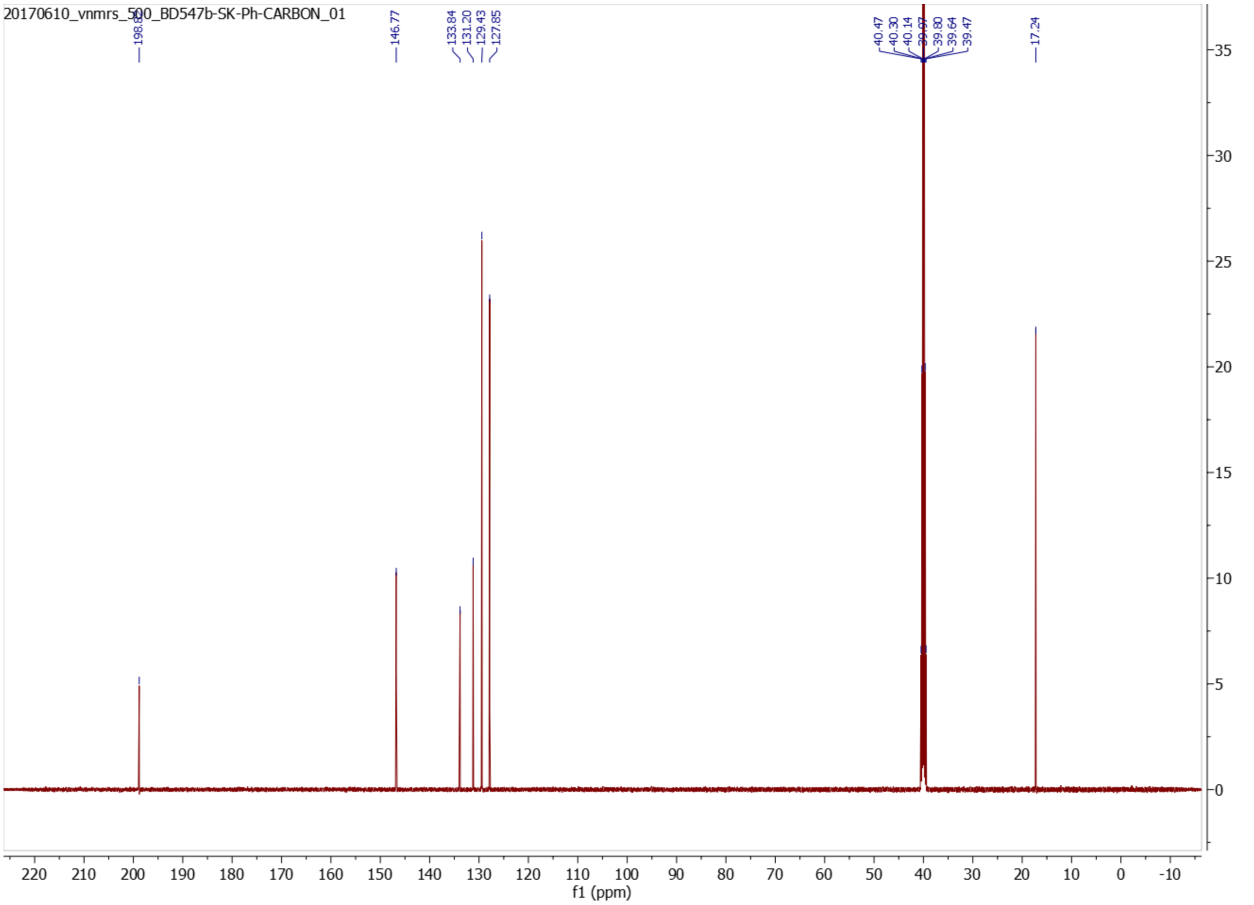

e Benzaldehyde *S*-ethyldithiocarbazate (9) ^{1}H NMR

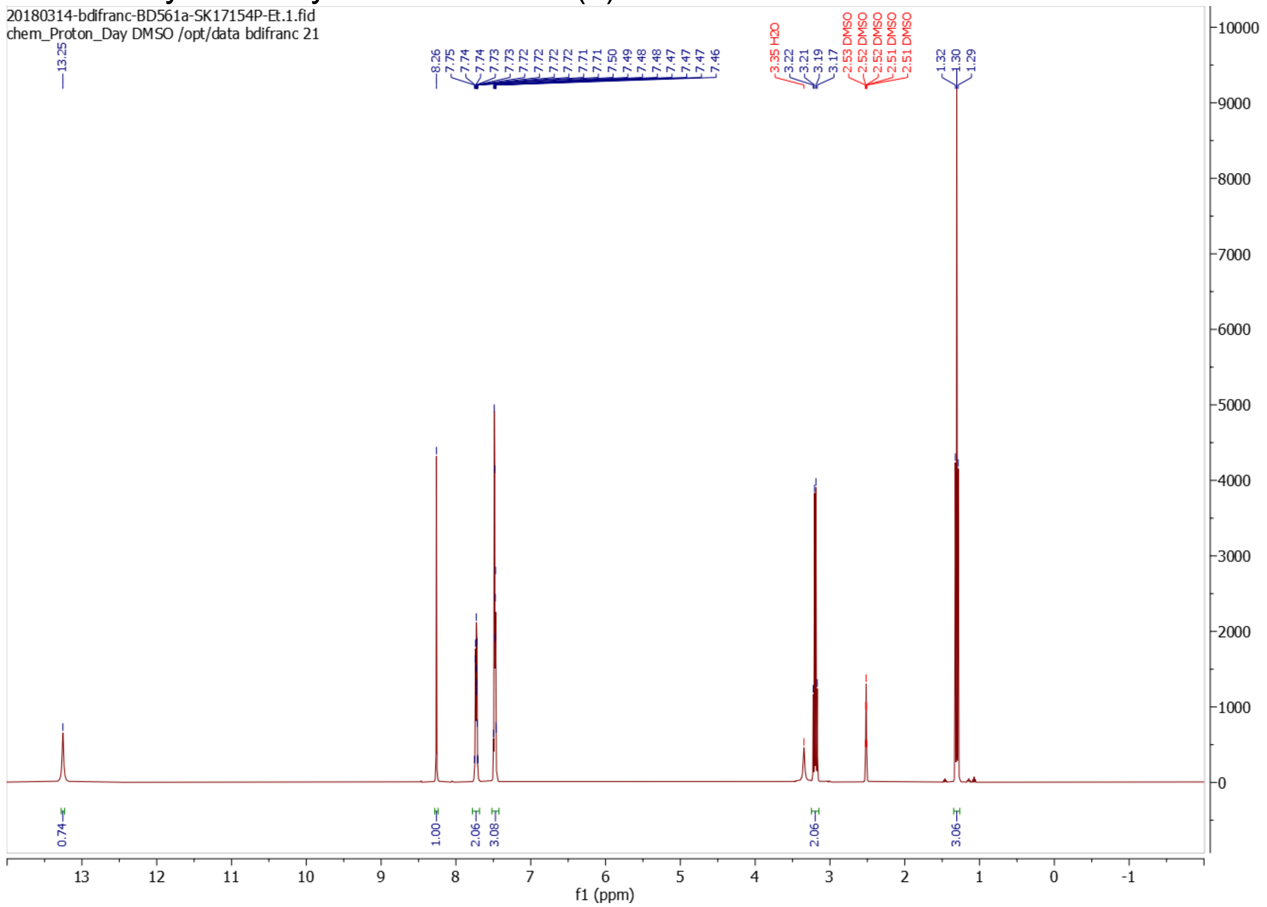

f Benzaldehyde *S*-ethyldithiocarbazate (9) ^{13}C NMR

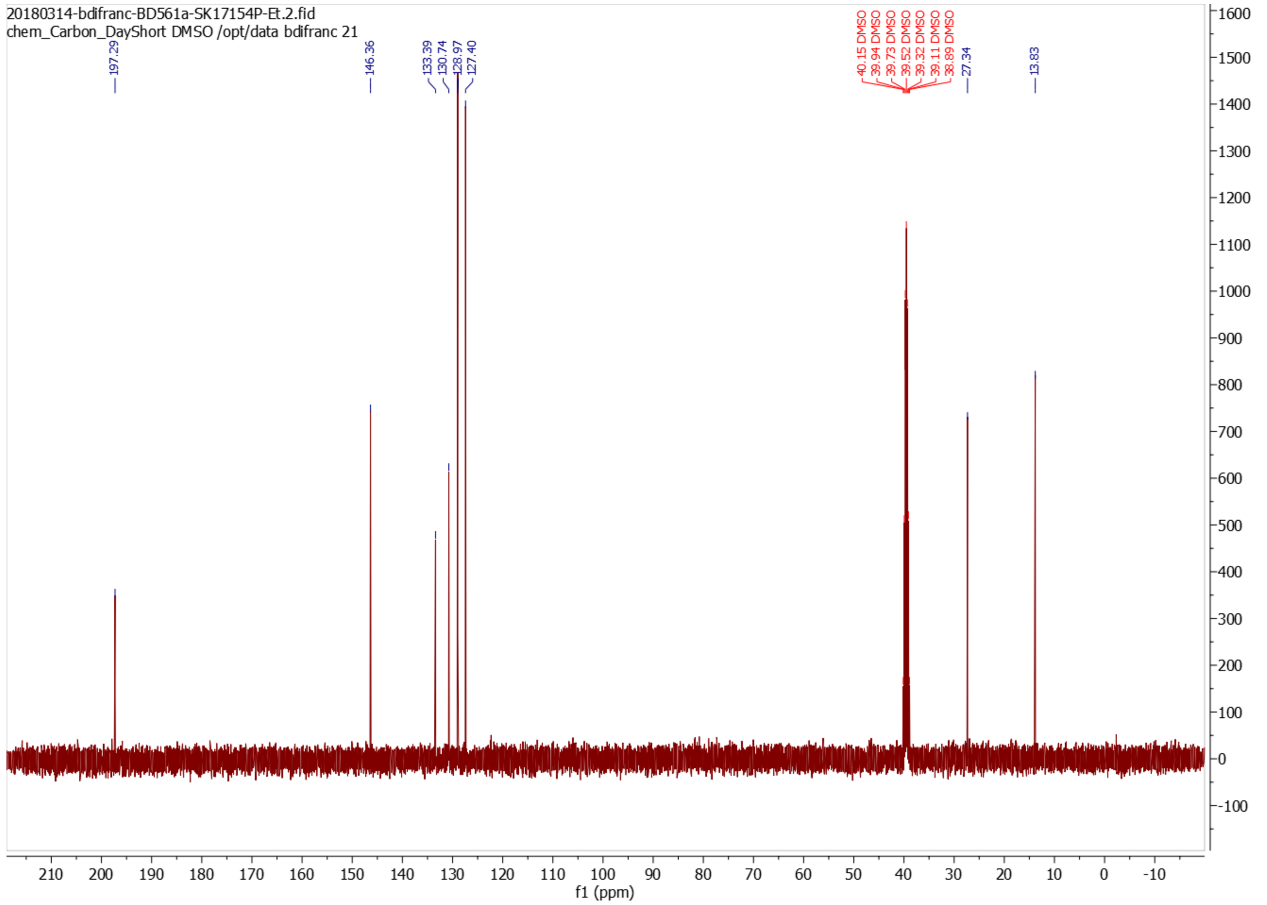

g Benzaldehyde *S*-benzyldithiocarbazate (10) ^{1}H NMR

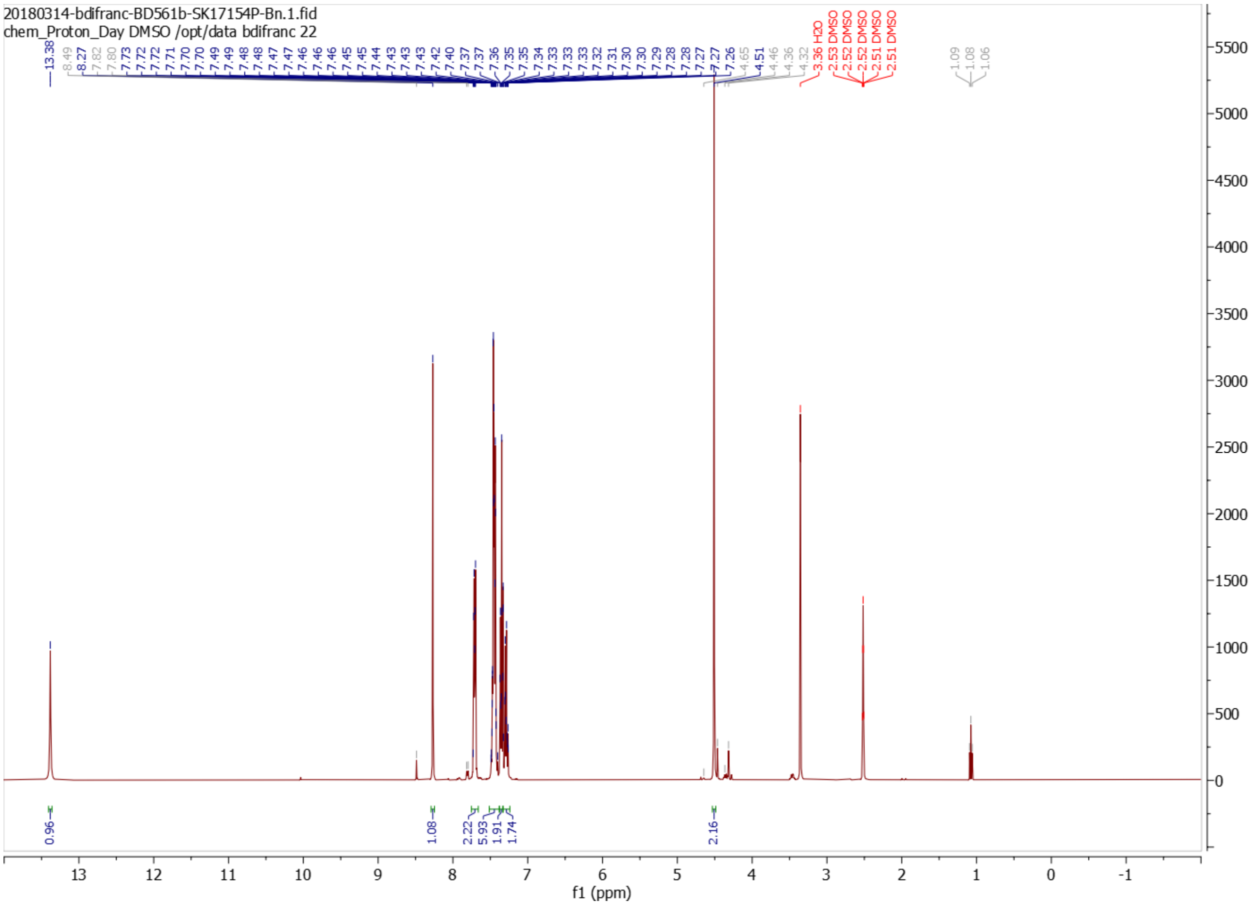

h Benzaldehyde *S*-benzyldithiocarbazate (10) ^{13}C NMR

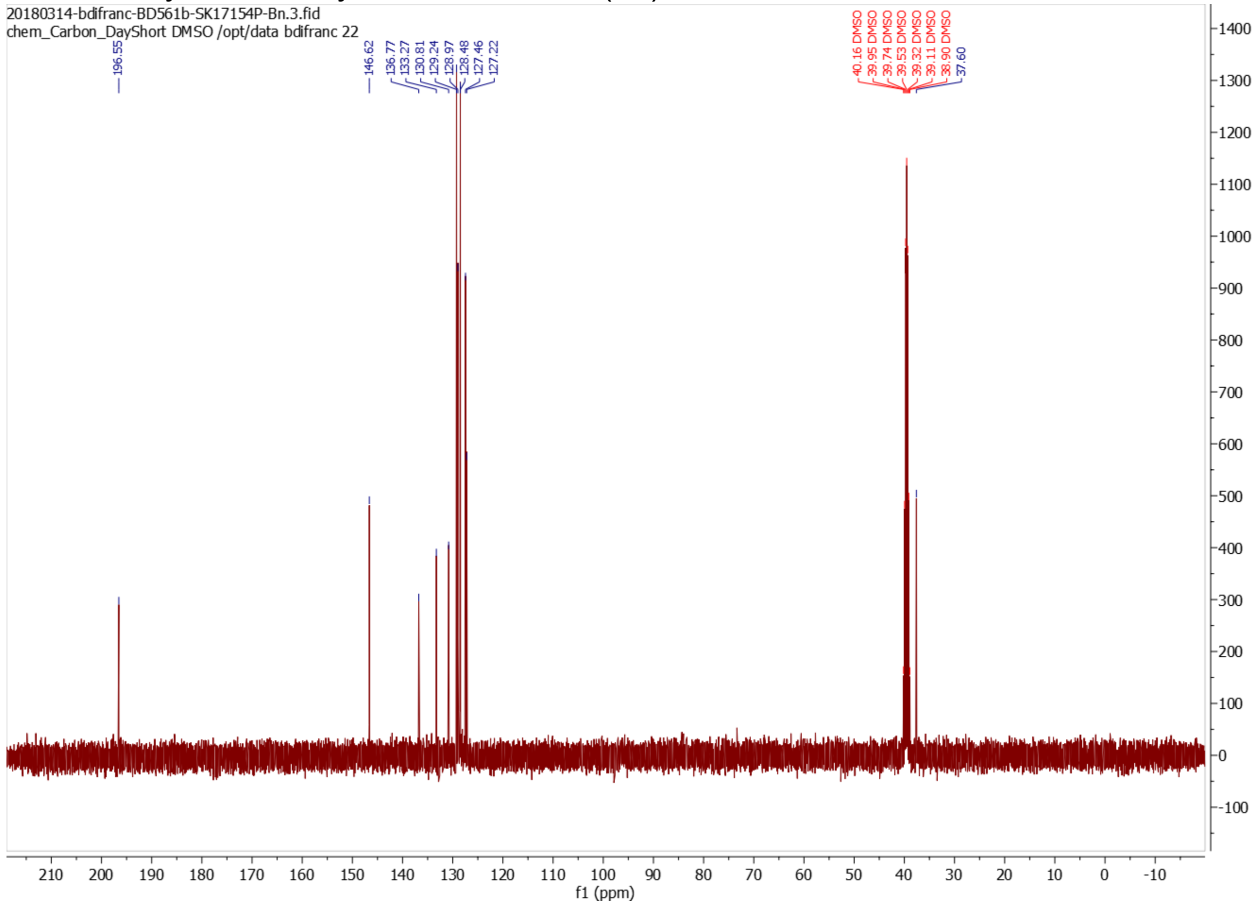

i

Benzaldehyde methylcarbazate (11) ^{1}H NMR

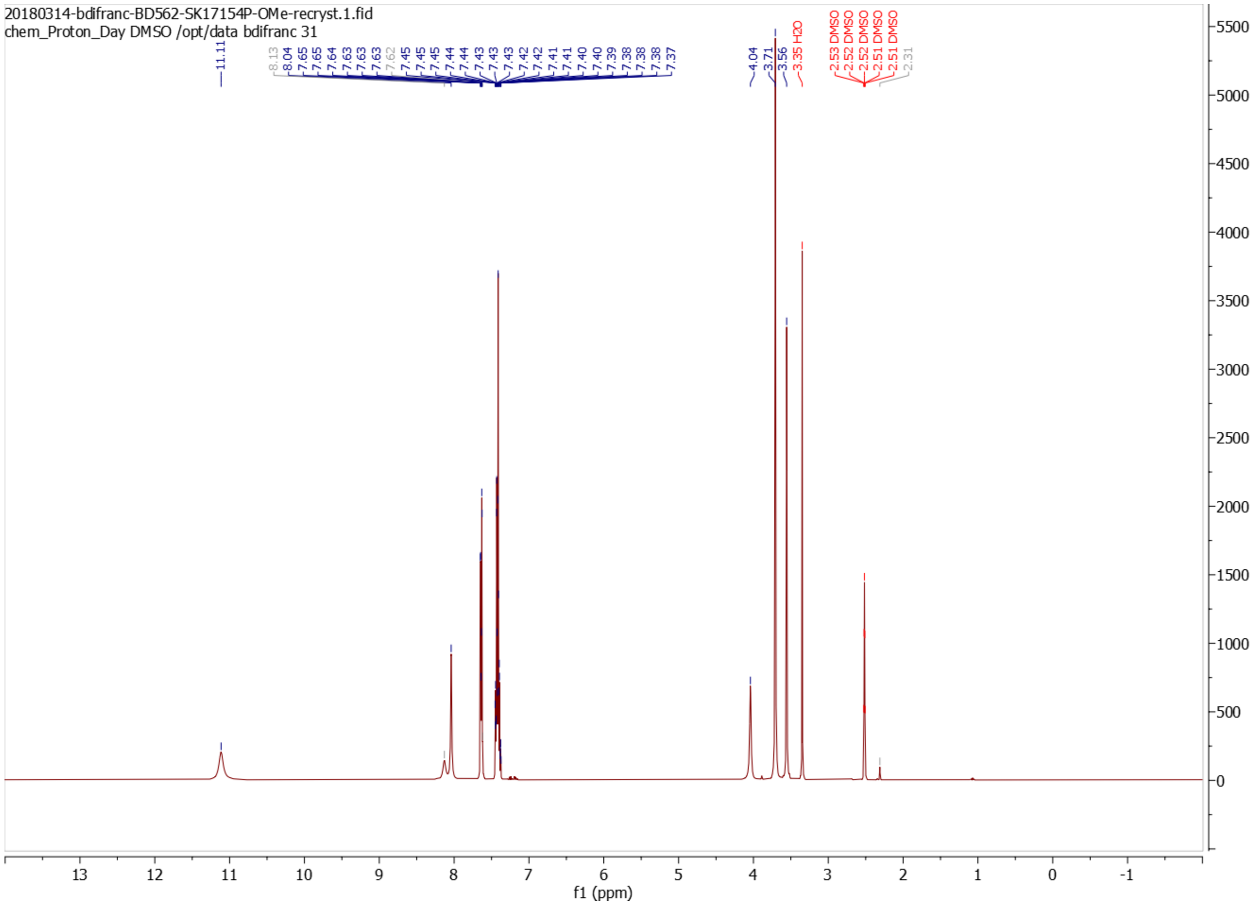

j

Benzaldehyde methylcarbazate (11) ^{13}C NMR

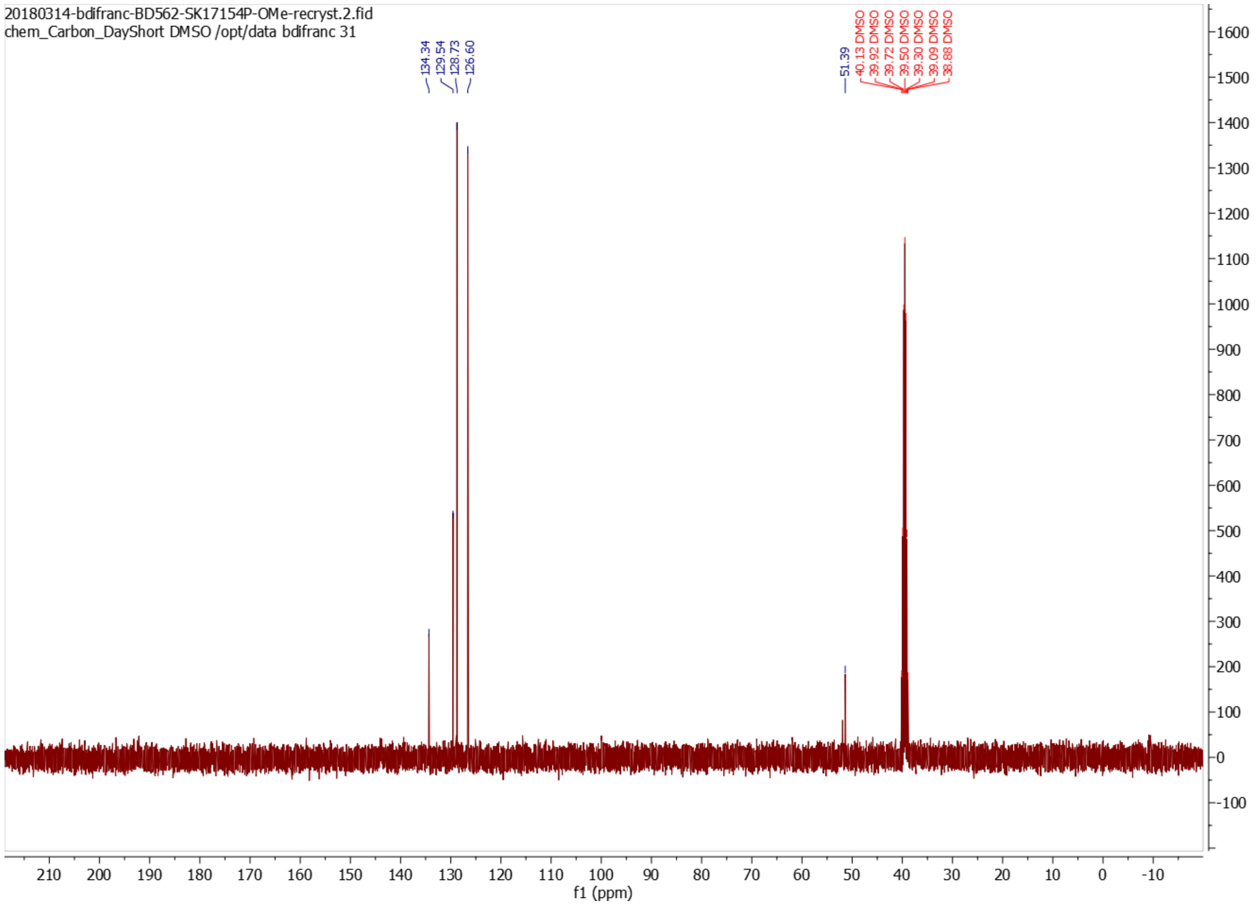

k Methyl 2-phenylethyl carbamodithioate (12) ^{1}H NMR

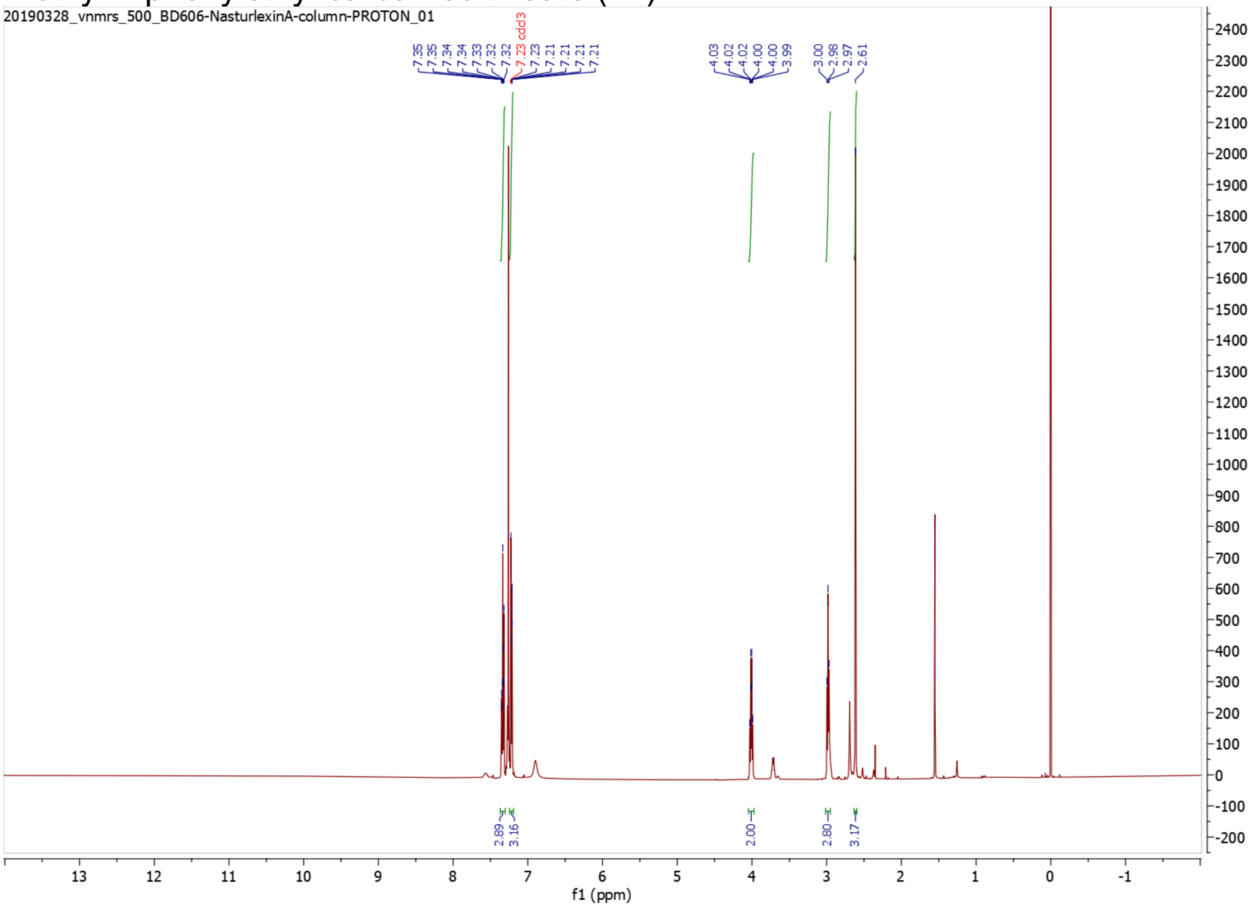

l Methyl 2-phenylethyl carbamodithioate (12) ^{13}C NMR

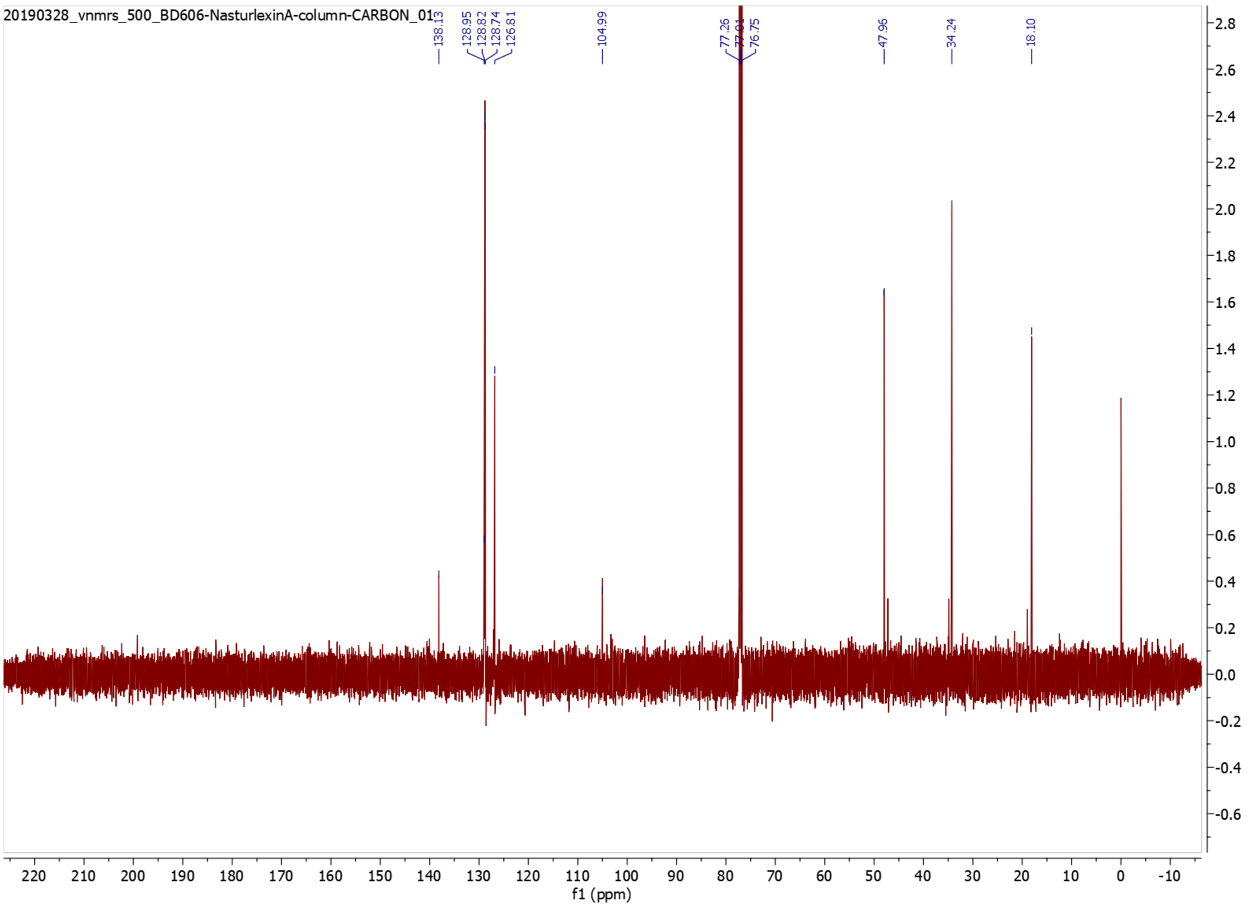

Supplement: Supplemental file 1 — Supplemental material. Download spectrum.00296-23-s0001.pdf, PDF file, 3.1 MB [file spectrum.00296-23-s0001.pdf]
